# Polycomb-mediated genome architecture enables long-range spreading of H3K27 methylation

**DOI:** 10.1073/pnas.2201883119

**Published:** 2022-05-26

**Authors:** Katerina Kraft, Kathryn E. Yost, Sedona E. Murphy, Andreas Magg, Yicheng Long, M. Ryan Corces, Jeffrey M. Granja, Lars Wittler, Stefan Mundlos, Thomas R. Cech, Alistair N. Boettiger, Howard Y. Chang

**Affiliations:** ^a^Center for Personal Dynamic Regulomes, Stanford University School of Medicine, Stanford, CA 94305;; ^b^Department of Genetics, Stanford University, Stanford, CA 94305;; ^c^Research Group of Development and Disease, Max Planck Institute for Molecular Genetics, 14195 Berlin, Germany;; ^d^Institute for Medical and Human Genetics, Charité Universitätsmedizin, 10117 Berlin, Germany;; ^e^HHMI, University of Colorado, Boulder, CO 80309;; ^f^Department of Biochemistry, University of Colorado, Boulder, CO 80309;; ^g^BioFrontiers Institute, University of Colorado, Boulder, CO 80309;; ^h^Department of Developmental Genetics, Max Planck Institute for Molecular Genetics, 14195 Berlin, Germany;; ^i^Department of Developmental Biology, Stanford University, Stanford, CA 94305;; ^j^HHMI, Stanford University School of Medicine, Stanford, CA 94305

**Keywords:** 3D genome, heterochromatin, epigenetic silencing, Polycomb-group proteins, RNA-mediated Polycomb loops

## Abstract

Polycomb-group proteins play critical roles in gene silencing through the deposition of histone H3 lysine 27 trimethylation (H3K27me3) and chromatin compaction. This process is essential for embryonic stem cell (ESC) pluripotency, differentiation, and development. Polycomb repressive complex 2 (PRC2) can both read and write H3K27me3, enabling progressive spreading of H3K27me3 on the linear genome. Long-range Polycomb-associated DNA contacts have also been described, but their regulation and role in gene silencing remain unclear. Here, we apply H3K27me3 HiChIP, a protein-directed chromosome conformation method, and optical reconstruction of chromatin architecture to profile long-range Polycomb-associated DNA loops that span tens to hundreds of megabases across multiple topological associated domains in mouse ESCs and human induced pluripotent stem cells. We find that H3K27me3 loop anchors are enriched for Polycomb nucleation points and coincide with key developmental genes. Genetic deletion of H3K27me3 loop anchors results in disruption of spatial contact between distant loci and altered H3K27me3 *in cis*, both locally and megabases away on the same chromosome. In mouse embryos, loop anchor deletion leads to ectopic activation of the partner gene, suggesting that Polycomb-associated loops control gene silencing during development. Further, we find that alterations in PRC2 occupancy resulting from an RNA binding–deficient EZH2 mutant are accompanied by loss of Polycomb-associated DNA looping. Together, these results suggest PRC2 uses RNA binding to enhance long-range chromosome folding and H3K27me3 spreading. Developmental gene loci have unique roles in Polycomb spreading, emerging as important architectural elements of the epigenome.

## Significance

The relationship between long-range Polycomb-associated chromatin contacts and the linear propagation of histone H3 lysine 27 trimethylation (H3K27me3) by Polycomb repressive complex 2 (PRC2) is not well-characterized. Here, we nominate a role for developmental loci as genomic architectural elements that enable long-range spreading of H3K27me3. Polycomb-associated loops are disrupted upon loss of PRC2 binding and deletion of loop anchors results in alterations of H3K27me3 deposition and ectopic gene expression. These results suggest that Polycomb-mediated genome architecture is important for gene repression during embryonic development.

Regulation of gene expression is crucial for a myriad of biological processes, including embryonic development, tissue homeostasis, and dosage compensation (1–5). Gene silencing mediated by Polycomb repressive complexes 1 and 2 (PRC1 and PRC2) allows for temporal and tissue-specific control of gene expression during development, with aberrant regulation leading to cancer and congenital disorders (6, 7). Pluripotent stem cells, including both mouse embryonic stem cells (mESCs) and human induced pluripotent stem cells (iPSCs), utilize the repressive histone H3 lysine 27 trimethylation (H3K27me3) mark deposited by PRC2 to suppress cell type–specific expression programs, maintain pluripotency, and prime stem cells for differentiation into many lineages.

Polycomb-group proteins (PcGs) and their target genes are evolutionarily conserved (8), but how Polycomb is recruited to target genes is not fully understood. Polycomb response elements (PREs), a complex DNA element, underlie recruitment of PcGs in *Drosophila melanogaster*. In mammalian genomes, hypomethylated CG bases known as CpG islands may function similar to *Drosophila* PREs (9, 10) and there is some evidence that Polycomb recruitment and spreading occur within three-dimensional (3D) genome structures in addition to local spreading of H3K27me3 (2, 3, 11, 12). Several studies have revealed that PRC1 and PRC2 establish long-range interactions in various cell types and organisms (3, 11, 13–18). However, the relationship between PcGs, 3D chromatin landscape, and gene silencing remains unclear. Polycomb-associated interactions occur in the larger context of genome architecture and chromatin modifications, including DNA methylation (12, 19) and cohesin-dependent folding (2), but there is emerging evidence that Polycomb can aggregate in a cohesin-independent manner (20). Polycomb binding to both coding and noncoding RNA has been shown to be vital to PcG recruitment (21, 22). RNA has multiple effects on PRC2, capable of both inhibiting PRC2 enzymatic activity and evicting PRC2 from chromatin (23–25), likely helping Polycomb to sense and avoid transcriptionally active genes. Disruption of RNA binding to EZH2, the methyltransferase subunit of PRC2, alters PRC2 recruitment to chromatin in iPSCs, resulting in reduced H3K27me3 at target genes (26). As PcGs interact with chromatin to induce compaction and mediate long-range interactions of silenced genomic regions, disruption of PRC2 recruitment via loss of RNA binding to EZH2 may alter 3D genome architecture. However, the role of PRC2 recruitment in the establishment and maintenance of chromatin architecture, in particular long-range genomic contacts (19) and the relation to linear H3K27me3 propagation and gene silencing, is not well-known. Here, we apply H3K27me3 HiChIP (27) and optical reconstruction of chromatin architecture (ORCA) (28) combined with genetic perturbations of H3K27me3-associated loop anchors and EZH2 binding to investigate the mechanism by which long-range Polycomb-associated genomic contacts are established and their role in propagation of H3K27me3 to distant sites.

## Results

**Fig. 1.**
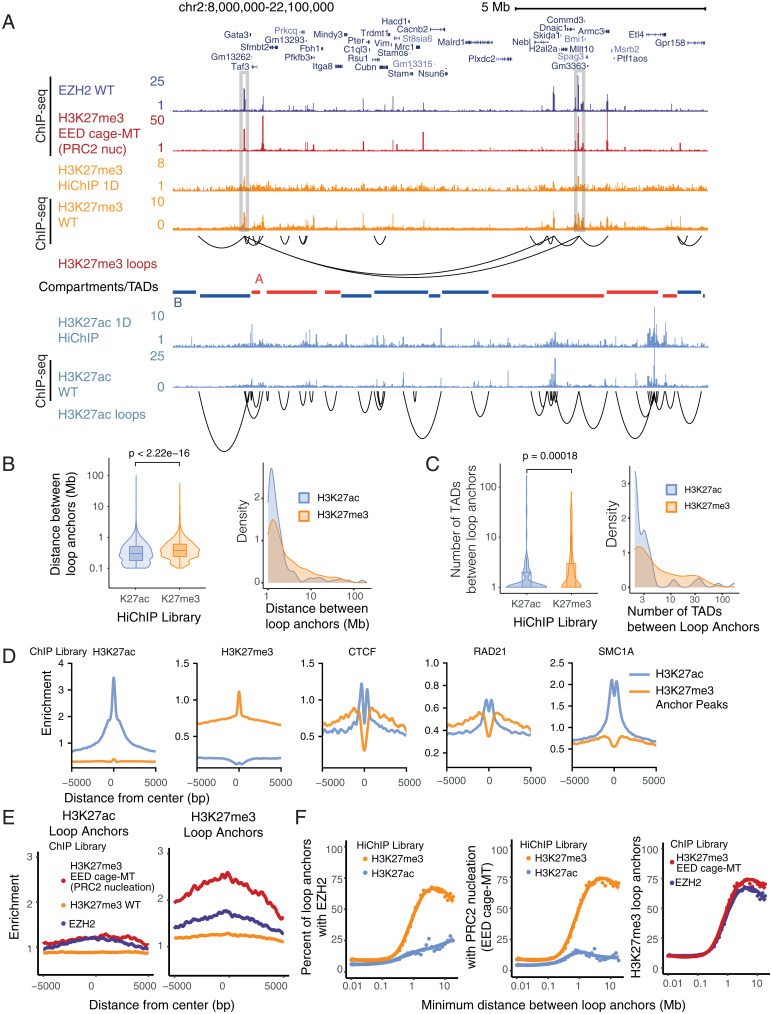
HiChIP identifies long-range Polycomb-associated interactions in mESCs. (*A*) HiChIP 1D signal enrichment and high-confidence HICCUPS loops for H3K27me3 and H3K27ac HiChIP at the *Gata3* locus (*n* = 1). H3K27me3, H3K27ac, and EZH2 ChIP-seq signals are shown for comparison. TADs from mESC Hi-C data colored by A/B compartment status are shown below. Selected regions with strong enrichment of H3K27me3 1D signal at long-range loops are highlighted. (*B* and *C*) Density and violin plots for the (*B*) distance between H3K27me3- and H3K27ac-associated loop anchors and (*C*) number of TADs between loop anchors. *P* values were calculated using Wilcoxon rank-sum test. (*D*) Signal enrichment for H3K27ac [ENCODE (31)], H3K27me3 [ENCODE (31)], CTCF (41), RAD21 (57), and SMC1A (57) ChIP-seq within a 10-kb window centered on H3K27ac (blue) or H3K27me3 (orange) ChIP-seq peaks [ENCODE (31)] in the respective HiChIP loop anchors. (*E*) Signal enrichment for EZH2, H3K27me3 WT, and EED MT ChIP-seq within a 10-kb window centered on H3K27ac or H3K27me3 HiChIP loop anchors, respectively. Units of enrichment were calculated as normalized ChIP-seq library depth per base pair per loop anchor. (*F*) Scatterplots illustrating the relation of minimum distance between loop anchors and the percentage of loop anchors overlapping EZH2 ChIP-seq peaks or EED MT H3K27me3 ChIP-seq peaks. The trend line represents a less smoothed fit with span 0.6. The shaded area represents 95% CI.

We performed HiChIP (27, 29) in mESCs using antibodies against two opposing histone modifications: repressive H3K27me3 deposited by PcG or enhancer-associated H3K27 acetylation (H3K27ac) (30). We observed 1D signal enrichment that recapitulated publicly available H3K27me3 and H3K27ac chromatin immunoprecipitation sequencing (ChIP-seq) datasets (31) (Fig. 1*A*) and enrichment of 1D HiChIP signal at corresponding H3K27me3 and H3K27ac ChIP-seq peaks (*SI Appendix*, Fig. S1*A*). We compared high-confidence loops called by HICCUPS (32) with those called by FitHiChIP (33), which additionally models the nonuniform coverage resulting from HiChIP enrichment. Over 90% of high-confidence HICCUPS loops were also detected by FitHiChIP (*SI Appendix*, Fig. S1*B*) and shared loops represented a higher-confidence subset of loops called by FitHiChIP (*SI Appendix*, Fig. S1 *C* and *D*). Therefore, we focused our analysis on 4,101 high-confidence H3K27me3-associated loops that were robustly detected over background and present on all chromosomes (*SI Appendix*, Fig. S1 *E* and *F*). Comparison of chromatin loops revealed that H3K27me3-associated loops bridge genomic distances spanning dozens of megabases, crossing significantly greater distances than H3K27ac-associated loops which are enriched at enhancer–promoter contact regions (*P* < 2.22 × 10^−16^, Wilcoxon rank-sum test; Fig. 1 *A* and *B*). Compared with enhancer loops marked by H3K27ac, the length distribution of H3K27me3-associated loops is asymmetric and has a long tail, in particular for loops that span over 1 Mb (Fig. 1*B*). For the top percentile of loops ranked by distance between loop anchors, the median distance between H3K27me3-associated loop anchors is 7.9 Mb compared with 2.0 Mb between H3K27ac-associated loop anchors.

As enhancer–promoter contacts tend to occur specifically within topologically associating domains (TADs) (16, 34, 35), we next examined how H3K27me3-associated loops behave in regard to previously known genome architecture units, TADs bound by CTCF, or larger-scale chromosome A/B compartments (32). We find that Polycomb loops can cross significantly more TADs than enhancer loops (*P* = 0.00018, Wilcoxon rank-sum test; Fig. 1*C*). For the top percentile of loops ranked by the number of TADs crossed, the median number of TADs crossed is 10.5 TADs for H3K27me3-associated loops compared with 4 TADs between H3K27ac-associated loop anchors. These results suggest that long-range H3K27me3-associated loops may be independent units of genome architecture from TADs, which is consistent with the observation that 3D PcG genome architecture domains behave differently from CTCF/cohesin–mediated domains (36). Further, we find that H3K27me3 peaks at H3K27me3-associated loops are depleted for CTCF and the cohesin subunit RAD21 and SMC1A occupancy relative to H3K27ac peaks at H3K27ac-associated loops (Fig. 1*D* and *SI Appendix*, Fig. S2*A*), suggesting that cohesin-CTCF may be more important for H3K27ac-associated loops and that other factors may mediate H3K27me3-associated loops.

Next, we sought to identify features defining H3K27me3-associated loop anchors, specifically protein occupancy or histone modifications that are enriched at anchor points. The PcG complex PRC2 that deposits the H3K27me3 modification consists of four core subunits: histone methyltransferase EZH2, H3K27me3-binding protein EED, architectural subunit SUZ12, and histone-binding protein RBBP4 (1). Previous studies have demonstrated that mutations in the cage ring of EED disrupt interactions with H3K27me3 and lead to defects in Polycomb spreading, leading to H3K27me3 restriction at strong PcG binding sites termed PRC2 nucleation points, from which the H3K27me3 mark spreads (37). We overlaid loop anchor points with H3K27me3 ChIP-seq from EED cage mutant mESCs (in which H3K27me3 signal is enriched at PRC2 nucleation points as PRC2 cannot spread) (37) and published EZH2 ChIP-seq data (38) and found enrichment of both Polycomb nucleation points and EZH2 occupancy over H3K27me3 signal at H3K27me3 loop anchors of varying loop strength (Fig. 1*E* and *SI Appendix*, Fig. S2*B*), in contrast to H3K27ac loop anchors and all H3K27me3 peaks including nonlooping peaks (Fig. 1*E* and *SI Appendix*, Fig. S2*C*). Further, we find that PRC2 nucleation sites are more likely to overlap H3K27me3 loop anchors relative to all H3K27me3 peaks excluding PRC2 nucleation sites (*SI Appendix*, Fig. S2*D*). Interestingly, we find that enrichment of EZH2 occupancy and nucleation points at H3K27me3 loop anchors increases with increasing loop distance and is substantially enriched compared with H3K27ac-associated loops (Fig. 1*F*). These data suggest that PRC2 complexes, especially those occupying nucleation points, may bring together distant genomic regions to establish long-range H3K27me3-associated chromatin loops capable of spanning multiple TADs and compartments.

Given the role of PRC1 complexes in long-range genomic interactions (2, 13, 39, 40), we also examined enrichment of PRC1 subunit occupancy at H3K27me3 loop anchors (*SI Appendix*, Fig. S3). We find that PRC1 components such as Ring1B are also enriched at H3K27me3-associated loop anchors, suggesting that both PRC1 and PRC2 complexes are enriched at these long-range contacts. Prior studies (19, 40) have identified similar Polycomb-associated long-range DNA loops by overlapping ChIP-seq of PRC2 or PRC1 subunits, respectively, with Hi-C data. The addition of H3K27me3 HiChIP data in this work demonstrates that 1) the H3K27me3 modification is present on the same chromatin fiber that is involved in long-range looping; and 2) Polycomb loops can be efficiently detected with H3K27me3 HiChIP at ∼1/10th the sequencing cost compared with Hi-C (*SI Appendix*, Fig. S4*A*).

**Fig. 2.**
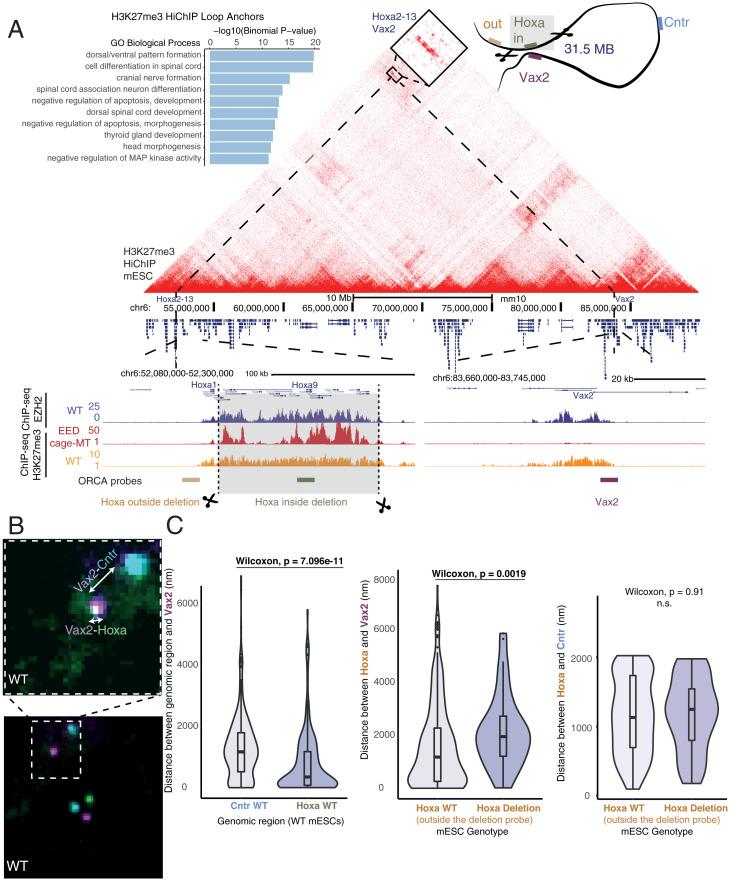
Deletion of the H3K37me3-associated loop anchor at the *Hoxa* cluster alters long-range 3D interactions. (*A*) Gene Ontology terms enriched at mESC H3K27me3-associated loop anchors (*Left*). H3K27me3 HiChIP contact matrix (10-kb resolution) visualizing Polycomb-associated interactions at the 40-Mb region encompassing the *Hoxa* cluster and *Vax2* in mESCs. ChIP-seq signal for WT EZH2 and H3K27me3 (WT and EED cage MT) and the position of ORCA probes are shown below. (*B*) ORCA imaging of three probes targeting *Hoxa*, *Vax2*, and control (Cntr) regions demonstrates the interaction between the *Hoxa* cluster with *Vax2* at the single-nucleus level in WT mESCs. Violin plots of the distance between *Vax2* and the *Hoxa* or *Cntr* probes (WT cells, *n* = 2,190; MT cells, *n* = 520). The *Hoxa* probe located within the Polycomb-associated loop anchor was used in WT mESCs. (*C*) Violin plots of the distance between *Hoxa* and *Vax2* (as measured by ORCA) for WT and *Hoxa* deletion mESCs (*Left*). Violin plots of the distance between *Hoxa* and *Cntr* (as measured by ORCA) for WT and *Hoxa* deletion mESCs (*Right*). As the *Hoxa* Polycomb-associated loop anchor is deleted in *Hoxa* deletion mESCs, a probe targeting a region adjacent to the *Hoxa* loop anchor was used for both WT and Hoxa deletion mESCs. Wilcoxon test was used for significance. n.s., not significant.

To determine which genes are involved in H3K27me3-associated loops, we performed Gene Ontology analysis for the nearest genes to loop anchors and found significant enrichment of developmentally associated processes with distinct ontology term enrichment compared with all H3K27me3 peaks excluding those at loop anchors (Fig. 2*A* and *SI Appendix*, Fig. S4*B*). While many of these loops, such as those at *Hoxa1*, *Hmx1*, and *Wnt6*, are also detected in deeply sequenced Hi-C (41), we observed strong enrichment for these contacts at lower sequencing depth in H3K27me3 HiChIP when comparing virtual 4C profiles (*SI Appendix*, Fig. S4*A*). Many key developmental genes encoding transcription factors (TFs) for cell-type specification and patterning, such as the *Hoxa* gene cluster, make long-range H3K27me3-associated loops with EZH2 occupancy at both anchors but PRC2 nucleation points occasionally only at one anchor (Fig. 2*A*). While we found at least one PRC2 nucleation point for 62% of H3K27me3-associated loops spanning over 1 Mb, 37% of these had a PRC2 nucleation point at only one anchor. We sought to characterize three different examples of these developmental gene-associated loops: 1) a previously described 31-Mb long-range loop between the *Hoxa* cluster and *Vax2* (3, 41) with EZH2 occupancy at both anchors but with only *Hoxa* as a PRC2 nucleation site (Fig. 2*A*); 2) a complex loop including several anchorpoints connecting *Nkx1-1*, *Hmx1*, and *Msx1* with EZH2 occupancy and nucleation points at all anchor points (*SI Appendix*, Fig. S5); and 3) a previously unobserved 3.8-Mb loop (42) connecting *Wnt6* and *Pax3* across the *Epha4* TAD with EZH2 occupancy and PRC2 nucleation points at both anchors (*SI Appendix*, Fig. S6).

To test the relationship between long-range looping and H3K27me3 spreading by PRC2, we used CRISPR-Cas9 editing to generate homozygous deletion alleles of loop anchors containing both PRC2 nucleation and EZH2 occupancy sites (Fig. 2*A*). To interrogate the effects of these deletions and provide an independent measurement of long-range H3K27me3-associated contacts, we monitored resulting genome architecture changes using ORCA, combining multiplexed DNA fluorescence in situ hybridization to sequentially image DNA loci at resolutions higher than the diffraction limit (28) (Fig. 2*B*). To enable ORCA following CRISPR-Cas9 editing, we designed probes to target both the original loop anchor, such as the *Hoxa* cluster, as well as a region adjacent to the deletion. Additional probes were placed at the *Vax2* loop anchor 31 Mb away and at a control region located at the midpoint of the two loop anchors in linear genomic distance. In order to fully remove the PRC2 nucleation and EZH2 occupancy sites, we deleted the majority of the *Hoxa* cluster from *Hoxa2* to *Hoxa13*, leaving *Hoxa1*, which is outside of the loop anchor detected by HiChIP. *Hoxa* cluster genes *Hoxa2* to *Hoxa13* included in the deletion are not expressed in mESCs so the effects of deletion should not be attributed to altered dosage of deleted genes. ORCA confirmed the presence of a 31-Mb loop identified by HiChIP in unedited cells, with 40% of cells positive for contact (<150 nm) between *Hoxa* and *Vax2* by high-resolution imaging (Fig. 2*B*). In cells where the *Hoxa* loop anchor was deleted, we observed a significant increase in the physical distance between *Vax2* and *Hoxa* (Fig. 2*C*). This supports the importance of the loop anchor which contains EZH2 occupancy and PRC2 nucleation points in establishing long-range contacts, which upon removal affects the spatial organization of regions outside of the original loop anchor.

**Fig. 3.**
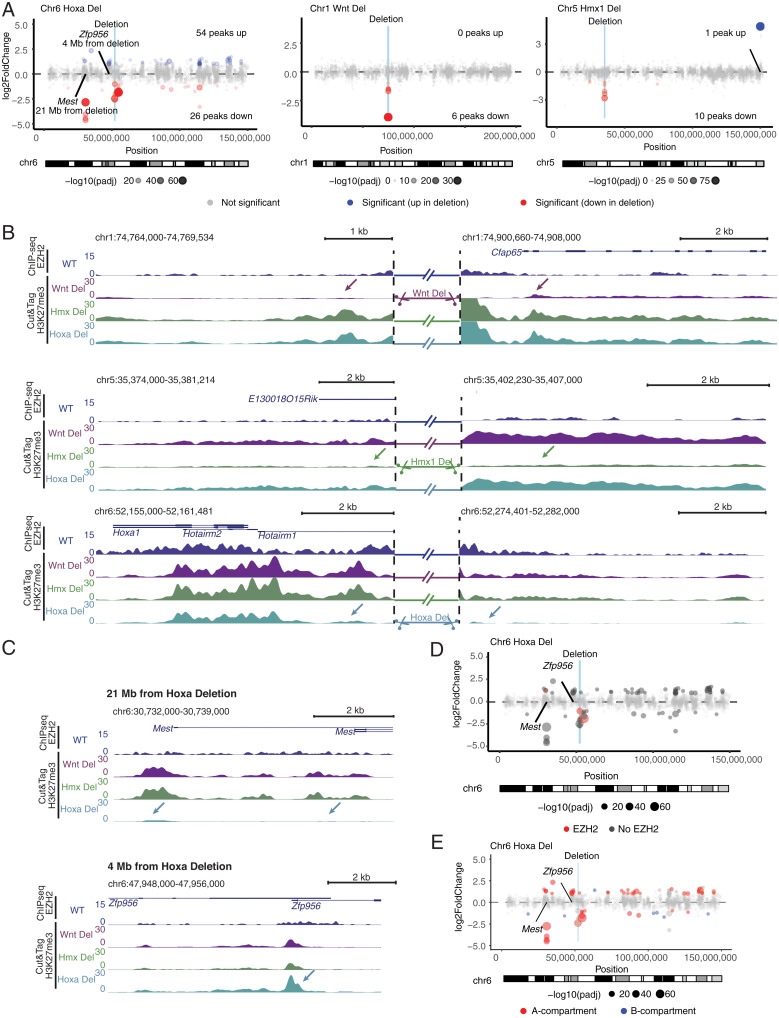
Deletion of Polycomb-associated loop anchors leads to both local and long-range changes in H3K27me3 modification *in cis*. (*A*) Scatterplots for the three different anchor point deletions (*Hoxa*, *Wnt*, *Hmx1*) illustrating the effects on altered H3K27me3 Cut&Tag signal versus the genomic position on the chromosome. Log_2_ fold changes (log_2_FCs) and *P* values (cutoff of absolute value log_2_FC > 1 and Benjamini–Hochberg–adjusted *P* value < 0.05 for significance) calculated in DESeq2 for each anchor point deletion clone (*n* = 3 replicates) relative to others. (*B*) Local changes in the vicinity of anchor point deletions are depicted by H3K27me3 Cut&Tag signal tracks. Arrows indicate significantly altered regions in MTs. EZH2 ChIP-seq signal in WT mESCs is shown above. Signal at deleted regions is omitted for clarity. (*C*) Long-range alterations in H3K27me3 resulting from the deletion of the *Hoxa* loop anchor, resulting both in down- and up-regulation (indicated with arrows). (*D* and *E*) Scatterplots as in *A* but showing the relation of H3K27me3 signal depending on (*D*) the occupancy of EZH2 at the altered site or (*E*) A/B compartment status.

We next asked if deletion of H3K27me3 loop anchors can affect spreading of the Polycomb-mediated H3K27me3 mark. We performed H3K27me3 Cut&Tag (cleavage under targets and tagmentation) (43) in mESCs with deletions for each loop anchor at the *Hoxa*, *Wnt6*, or *Hmx1* loci and identified genomic loci with differential H3K27me3 deposition, focusing on significantly altered sites *in cis* more likely to be directly impacted by loop anchor deletion (Fig. 3*A*). Because the *Hoxa* (*Hoxa2* to *Hoxa13)*, *Wnt6*, and *Hmx1* genes are not expressed in mESCs, the effects we observe are most likely to result from deleting the genomic region and not from eliminating expression of these genes. Our data showed reduced H3K27me3 adjacent to deletion breakpoints, consistent with loss of local Polycomb spreading (Fig. 3*B*). For the *Wnt6* and *Hmx1* deletions, alterations in H3K27me3 were largely restricted to local alterations in mESCs, although we observed significant differences in H3K27me3 at distant sites tens of megabases from the deletion site in the *Hoxa* deletion, with unaltered H3K27me3 sites in between (Fig. 3 *A* and *C*). To investigate the cause of such distant effects, we examined the relationship between EZH2 occupancy and found that sites which lose H3K27me3 lack intrinsic EZH2 occupancy (Fig. 3 *C* and *D* and *SI Appendix*, Fig. S7). These regions also tend to colocalize in the same compartment (Fig. 3*E* and *SI Appendix*, Fig. S7). Interestingly, we observed no significant changes in H3K27me3 at long-range contacts with deleted loop anchors identified by HiChIP, all of which have EZH2 occupancy. These data suggest an intriguing possibility that PRC2 nucleation sites such as the *Hoxa* gene cluster may distribute the repressive H3K27me3 mark to distant regions within the same compartment which lack intrinsic Polycomb binding. We predict that these interactions are dynamic and transient, as we did not identify significant looping interactions between the deleted nucleation sites and regions with loss of H3K27me3 in our HiChIP data. We also identified sites with increased H3K27me3 at local as well as distant sites (Fig. 3*C*), consistent with recent work demonstrating global redistribution of PcG complexes upon perturbations such as BAF depletion (44).

**Fig. 4.**
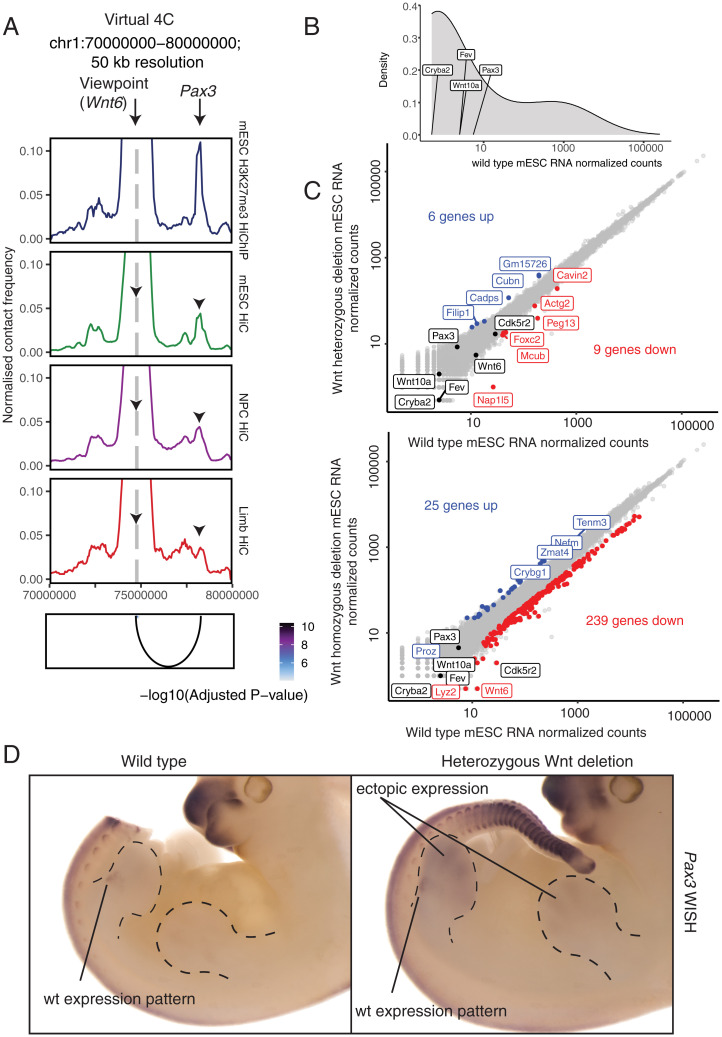
Heterozygous deletion of the *Wnt6* anchor results in derepression and ectopic expression of the loop partner *Pax3* in mouse E11.5 embryos in distal limb buds. (*A*) Virtual 4C interaction profile at the *Wnt6* promoter for mESC H3K27me3 HiChIP (this study), mESC Hi-C (41), NPC Hi-C (41), and E11.5 limb bud Hi-C (45), scaled by the number of filtered read pairs. (*B*) Density plot of normalized read counts for all RNA transcripts in WT mESCs with genes included in Wnt deletion and the anchor partner *Pax3* marked. (*C*) Scatterplot of normalized RNA-seq read counts for WT mESCs vs. Wnt heterozygous (*Top*) and homozygous (*Bottom*) deletion mESCs. Transcripts significantly higher following Wnt deletion (log_2_FC > 1 and *P*_adj_ < 0.05) are highlighted in blue and transcripts significantly lower following Wnt deletion (log_2_FC < −1 and *P*_adj_ < 0.05) are highlighted in red. Genes included in Wnt deletion and anchor partner *Pax3* are highlighted in black unless differentially expressed. (*D*) Pattern of forelimb expression of *Pax3* in an MT carrying heterozygous *Wnt10a*/*Wnt10* genomic deletion and endogenous pattern of expression by whole-mount in situ hybridization (WISH) at E11.5. WT expression is restricted to muscle precursor cells in the proximal limb bud. Note the gain of expression in the distal portion of the limb bud (the tail in the WT embryo is removed to stain embryos in the same well for proper control).

Given that H3K27me3 at distant sites could be maintained by local propagation of existing H3K27me3 marks in the mESC state, we hypothesized that functional consequences of distal Polycomb-mediated contacts may be more relevant in the context of differentiation. To address this possibility, we generated embryos from an edited mESC line with heterozygous deletion of *Wnt6*, the loop anchor partner of *Pax3* (Fig. 4*A* and *SI Appendix*, Fig. S6). Importantly, this loop is maintained during differentiation in both the developing limb bud and in differentiated neural progenitor cells (NPCs) (Fig. 4*A*) (41, 45). We observed minimal significant changes in gene expression following heterozygous and homozygous deletion of the *Wnt6* anchor in mESCs, both *in cis* and *in trans* (Fig. 4 *B* and *C*). In contrast, heterozygous deletion of the *Wnt6* anchor results in derepression and ectopic expression of the loop partner *Pax3* in mouse embryonic day 11.5 (E11.5) embryos in distal limb buds, demonstrating the in vivo relevance of this loop for gene silencing (Fig. 4*D*). This result suggests that long-range Polycomb-mediated genome organization maintains gene silencing at distal loci during development.

To determine if long-range Polycomb loops are conserved features across evolution, we performed H3K27me3 HiChIP in human iPSCs. We converted genomic coordinates for mESC H3K27me3-associated loop anchors to the human genome and found that ∼30% of high-confidence loops identified by H3K27me3 HiChIP in human iPSCs were shared with mouse ESCs, and conserved H3K27me3 loops in the human genome were enriched for similar developmental Gene Ontology terms as observed in mESCs (*SI Appendix*, Fig. S8*A*).

**Fig. 5.**
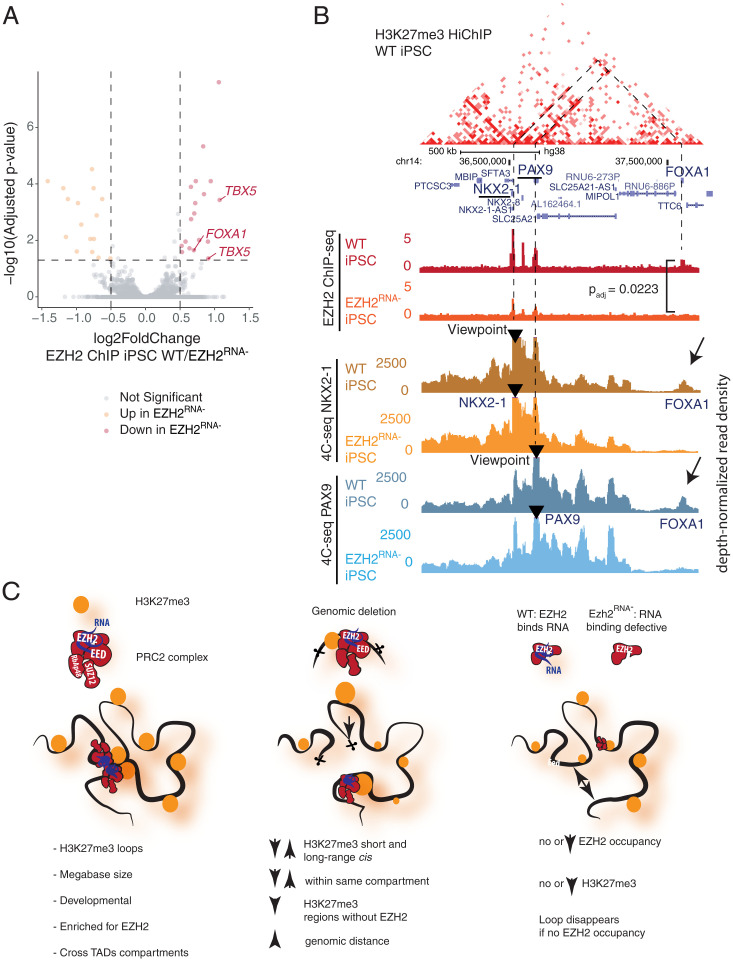
Altered Polycomb binding due to loss of RNA binding by EZH2 alters genome architecture in human iPSCs. (*A*) Volcano plot of differential EZH2 ChIP-seq peak signal in WT vs. *EZH2^RNA−^* iPSCs (26). Log_2_ fold changes and *P* values (cutoff of absolute value log_2_FC > 0.5 and Benjamini–Hochberg–adjusted *P* value < 0.05 for significance) were calculated in DESeq2. Points are labeled by the nearest gene relative to the peak. (*B*) WT H3K27me3 HiChIP contact matrix at the *NKX2-2*/*PAX9*/*FOXA1* locus with H3K27me3 and EZH2 ChIP-seq and 4C-seq shown as depth-normalized read density at *NKX2-1* and *PAX9* viewpoints in WT and *EZH2^RNA−^* iPSCs. Lost contact with *FOXA1* accompanied by loss of EZH2 binding highlighted with the Benjamini–Hochberg–adjusted *P* value for EZH2 ChIP-seq signal (WT/*EZH2^RNA−^* iPSCs) is shown. (*C*) Summary of the findings. In WT stem cells, Polycomb-associated H3K27me3 loops connect vast genomic distances spanning dozens of megabases, crossing TADs and A/B compartments. Deletion of anchor points leads to both local and distal changes of H3K27me spreading *in cis*, preferentially affecting regions which lack EZH2 occupancy and are located in the same compartment as the original anchor. RNA binding–deficient mutant EZH2 results in loss of looping at loci at sites with reduced EZH2 occupancy.

To examine the broader effects of PRC2 binding on genome architecture, we utilized an EZH2 variant with mutations in two regions (F32A, R34A, D36A, K39A; 489 to 494 PRKKKR to NAAIRS) that is deficient in RNA binding but otherwise has normal PRC2 complex formation and intact H3K27me3 methylase activity (26, 46). This RNA binding–deficient EZH2 mutant (*EZH2^RNA−^* hereafter) results in genome-wide alterations in Polycomb binding and H3K27me3 deposition with more pronounced effects at specific sites such as *TBX5* and *FOXA1* (Fig. 5*A*), as described in a prior study (26). To ask how EZH2’s promiscuous RNA binding may contribute to H3K27me3-associated chromatin architecture, we performed H3K27me3 HiChIP in wild-type (WT) and *EZH2^RNA−^* iPSCs. *EZH2^RNA−^* iPSCs had significantly reduced contacts at Polycomb anchor sites at key developmental loci, including *HOX*, *PAX*, *NKX*, and *TBX* (*SI Appendix*, Fig. S8*B*). Because *EZH2^RNA−^* iPSCs have decreased H3K27me3 at these same loci, resulting changes in HiChIP signal can be attributed to either loss of H3K27me3 modification or loss of 3D contact. Therefore, we performed 4C-seq (47), a targeted DNA proximity assay not dependent on ChIP enrichment, at viewpoints in regions with differential contact identified by HiChIP, including an H3K27me3-associated loop that spans the adjacent *PAX9* and *NKX2-1* locus to the *FOXA1* locus ∼500 kb away. *PAX9-NKX2-1* shows strong EZH2 occupancy while *FOXA1* has modest EZH2 occupancy in WT cells (Fig. 5*B*). In *EZH2^RNA−^* iPSCs, *PAX9-NKX2-1* no longer comes into proximity with *FOXA1* as shown by 4C-seq with two independent viewpoints and primer sets, and concordantly *FOXA1* loses EZH2 occupancy (Fig. 5*B*). Similarly, 4C-seq analysis of the *TBX3* and *TBX5* loci shows a similar deficit of the *EZH2^RNA−^* mutant in Polycomb-mediated chromosome looping and spread of EZH2 occupancy from the putative nucleation point (*TBX3*) to its loop partner (*TBX5*) but minimal effects on chromosome looping at sites such as the *LHX5* locus which maintains EZH2 occupancy (*SI Appendix*, Fig. S8*C*). These results suggest that RNA binding by EZH2 is required to drive long-range chromosome looping and spread Polycomb occupancy to distant loci.

## Discussion

Here, we apply H3K27me3 HiChIP to identify long-range Polycomb-associated loops among developmental genes, and demonstrate that local or global loss of Polycomb binding can mediate long-range effects on H3K27me3 deposition and genome architecture. Polycomb loops link H3K27me3-modified loci from the same chromatin compartment that are separated by tens to hundreds of megabases on the linear chromosome, and they appear orthogonal to the ∼1-Mb-size TADs based on CTCF and cohesin, indicating an intermediary structure in the hierarchical organization of 3D genome folding. This finding is consistent with studies that conclude that PRC1-associated interactions are independent of CTCF and TADs (2, 40). While changes in enzymatic activity of PRC1 do not impair these interactions, our study demonstrates loss of Polycomb-associated interactions following disruption of the PRC2 RNA-binding domain, suggesting there may be distinct roles for PRC1 and PRC2 in regulation of genome architecture (40). Zhang et al. recently reported long-range chromosomal loops in hematopoietic stem cells marked by nadirs of DNA methylation and high H3K27me3 (19); thus, Polycomb loops may be a conserved feature of embryonic and adult tissue stem cells. While prior studies have demonstrated the involvement of PRC1 in maintenance of these long-range contacts (2, 13, 39, 40), our results demonstrate alterations in both PRC2 occupancy and long-range interactions following disruption of RNA-binding mutation by PRC2 component EZH2 in iPSCs and also nominate a role for PRC2 in maintaining long-range interactions. While the relative contributions of PRC1 and PRC2, as well as cross-talk between these complexes, require further study, we acknowledge that both PRC1 and PRC2 play an important role in establishment of genome organization.

Recent work by Ngan et al. also nominates the role of PRC2 in genome organization and gene silencing and demonstrates that homozygous deletion of PRC2-bound silencers can lead to gene expression changes of interacting genes *in cis* (18). While Ngan et al. described local effects within 500 kb, our in vivo results demonstrate ectopic gain in gene expression in the developing limb bud at an interacting gene located 3.8 Mb away following heterozygous deletion of a PRC2-bound loop anchor. Additionally, by leaving one allele intact, we avoid potential loss-of-function effects that could occur *in trans*. Together, these results support an important role for PRC2 in long-range chromatin interaction and gene silencing in vivo.

Our results also suggest a view of developmental gene loci as architectural elements of the epigenome, nucleating and spreading H3K27me3—a role analogous to centromeres and telomeres that mediate position effect variegation through the spread of H3K9me3 (48). Because all three of the TF loci we deleted are not transcribed in ESCs, the effects of locus deletion on long-range H3K27me3 deposition are likely due to architectural roles of these loci as noncoding regulatory DNA elements. These effects should be considered when investigators interpret large-scale deletions of *Hox* and other developmental gene loci. We find that EZH2 occupancy, specifically at sites mediated by RNA binding, is essential in establishment of long-range genomic contacts and spreading of H3K27me3, potentially connecting widely separated chromatin regions (Fig. 5*C*). PRC2 binds thousands of RNAs on chromatin (22, 49, 50) and inhibition of EZH2–RNA interactions results in genome-wide reductions in EZH2 occupancy, altered H3K27me3 modification, and differentiation defects (26, 51).

Many developmental TF loci encode positionally conserved noncoding RNA transcripts (52), which may facilitate PRC2 spreading and enforcement of chromosome contacts. While the specific RNA species involved and detailed recruitment mechanisms should be addressed in future studies, the results of this study suggest that Polycomb-associated contacts may be important for proper gene regulation during development.

## Methods

A full description of materials and methods is available in *SI Appendix*.

### mESC Culture.

mESCs were cultured in ESC medium containing knockout Dulbecco’s modified Eagle’s medium, 15% fetal calf serum, and 1,000 U/mL leukemia inhibitory factor.

### CRISPR-Cas9–Engineered Structural Variants in mESCs and Transgenic Animals.

mESCs carrying the desired deletions were generated according to the CRISPR-Cas–induced structural variant (CRISVar) protocol (53). Briefly, per structural variant, two single-guide RNAs (sgRNAs) were designed using the “CRISPR guides” design tool of Benchling (https://www.benchling.com/), picking the guides showing the best off-target score. WT G4 mESCs (129/Sv × C57BL/6 F1 hybrid background) (54) were cotransfected with the two respective sgRNA-pX459 vectors using the FuGENE HD transfection reagent (Promega) following the manufacturer’s instructions. Individual ESC clones were screened for deletions via PCR and copy-number variation (CNV) qPCR and verified by PCR amplification and Sanger sequencing of the CRISPR breakpoint. Sequences of sgRNAs, CRISPR breakpoints, and genotyping PCR and CNV qPCR primers are listed in *SI Appendix*, Table S1. Embryos from mESCs were generated by tetraploid complementation (55). For each structural variation, at least two independent clones were aggregated. Genotyping was performed by PCR analysis. Guide primers, genotyping primers, and breakpoint coordinates are summarized in *SI Appendix*, Table S1.

### In Situ Hybridization.

In situ hybridization was performed according to standard protocols. The probe from *Pax3* was generated by PCR amplification using mouse limb complementary DNA. All animal procedures were in accordance with institutional, state, and government regulations (Regional Office for Health and Social Affairs, Berlin, Germany).

### Human iPSC Culture.

WT and mutant (MT) iPSC clones were obtained from the T.R.C. lab; MT iPSC clones were generated by Y.L. from the original WTC-11 iPSC from the Coriell Institute (GM25256, deposited by Bruce R. Conklin, Gladstone Institute, University of California, San Francisco, CA). For regular culture and passaging, cells were maintained in Essential 8 Flex medium (Thermo Fisher, A2858501) using vitronectin (Thermo Fisher, A14700) as the coating material and the medium was changed every other day until ready for passaging. To passage the cells, 5- to 10-min incubation with 0.5 mM ethylenediaminetetraacetate acid (EDTA) in phosphate-buffered saline (PBS) after a PBS wash step facilitated detachment and cells were split with a 1:6 (up to 10) ratio into new culture dishes coated with vitronectin. Cells were cryopreserved in Essential 8 Flex medium with 10% dimethyl sulfoxide for long-term storage.

### HiChIP.

Cells (5 × 10^6^) were fixed in 2% formaldehyde for 10 min at room temperature (RT). HiChIP was performed as previously described (27) using antibodies against H3K27me3 (Millipore Sigma, 07-449) and H3K27ac (Active Motif, 39133) with the following optimizations (29): sodium dodecyl sulfate treatment at 62 °C for 5 min; restriction digest for 15 min; no heat inactivation of restriction enzyme, instead a wash of nuclei twice with 1× restriction enzyme buffer; biotin fill-in reaction incubation at 37 °C for 15 min; and ligation at room temperature for 2 h.

### RNA-Seq.

For the analysis of differential gene expression, mESCs were directly lysed or microdissected, and homogenized using a syringe, respectively. RNA extraction and stranded messenger RNA (mRNA) library preparation were performed according to the manufacturers’ instructions using the Qiagen RNeasy Mini Kit and the KAPA mRNA Hyper Prep Kit, respectively. Each condition for WT or MT samples was sequenced in biological triplicates using Illumina HiSeq technology according to standard protocols.

### 4C-Seq.

The 4C-seq libraries were generated from fixed cells as described previously (47). *Hind*III (6-bp cutter) was used as a primary restriction enzyme. *Nla*III was used as a secondary restriction enzyme. For each viewpoint, a total of 1.6 mg of each library was amplified by PCR (primer sequences can be found in *SI Appendix*, Table S1). Samples were sequenced 2 × 75 bp with Illumina HiSeq 4000 technology according to standard protocols.

### Cut&Tag.

Cut&Tag experiments were performed according to Kaya-Okur et al. (43). In short, cells were harvested with Accutase and aliquots of 100,000 cells were conjugated to 10 µL activated concanavalin A–coated beads (Bangs Laboratories) per sample. Primary antibody incubation was performed for 2 h at RT in 100 µL antibody buffer (20 mM Hepes⋅KOH, pH 7.5, 150 mM NaCl, 0.5 mM spermidine, 0.05% digitonin, 2 mM EDTA, 0.1% bovine serum albumin, 1× protease inhibitors) and either 1 µL H3K27me3 (Active Motif, 61017) or immunoglobulin G (IgG) (abcam, ab6709) antibody (1:100). Incubation of the secondary antibody was performed for 1 h at RT with either rabbit anti-mouse (abcam, ab46540, for H3K27me3) or guinea pig anti-rabbit antibody (antibodies-online, ABIN101961, for IgG) in a 1:100 dilution in Dig-Wash buffer (20 mM Hepes⋅KOH, pH 7.5, 150 mM NaCl, 0.5 mM spermidine, 0.05% digitonin, 1× protease inhibitors). The pA–Tn5 adapter complex (in-house-made batch) was used at a 1:300 dilution in Dig-300 buffer (20 mM Hepes⋅KOH, pH 7.5, 300 mM NaCl, 0.5 mM spermidine, 0.01% digitonin, 1× protease inhibitor) and incubated with the samples for 1 h at RT. After the transposition reaction (1 h at 37 °C) and reverse-cross-linking (overnight at 37 °C followed by inactivation of proteinase K at 70 °C for 20 min), samples were purified using the Zymo ChIP DNA Clean and Concentrator Kit according to the manufacturer’s instructions. PCR was performed with i5/i7 Nextera index primers and NEBNext Hifi 2× PCR Master Mix, with a total of 14 cycles. Post-PCR cleanup was carried out by adding a 0.9× volume of Ampure XP beads and elution in 20 µL of ultrapure H_2_O.

### ORCA Imaging.

The primary probes tiling the regions of interest (*SI Appendix*, Table S1) were designed as previously described (28) with the modification of removing the fiducial labels on primary probes. Separate fiducial probes were designed corresponding to each chromosome of interest (*SI Appendix*, Table S1) spanning 200 kb of the chromosomes tiled by the experimental probes for image registration purposes. Probes were amplified from the oligopool (CustomArray), and amplified according to the protocol described (28, 56). In preparation for imaging, mESCs were collected and fixed in 4% paraformaldehyde (PFA) in 1× PBS for 10 min. Cells were then washed three times in 1× PBS and stored in 70% ethanol for up to 3 mo. Glass coverslips (40-mm; Bioptechs) were coated with poly-d-lysine for at least 1 h and then rinsed with 1× PBS to remove residue. A population of control and deletion mESCs was then plated directly onto the coverslip in two spatially distinct populations and allowed to dry for 7 to 10 min. Once cells were dried and adhered to the slide, hybridization and imaging were performed as previously described (28). For primary probe hybridization, cells were permeabilized for 10 min with 0.5% Triton-X in 1× PBS, and the DNA was then denatured by treatment with 0.1 M HCl for 5 min. Two micrograms of primary probes in hybridization solution was then added directly to the cells, placed on a heat block at 90 °C for 3 min, and incubated overnight at 42 °C in a humidified chamber. Prior to imaging, the samples were postfixed for 1 h in 8% PFA + 2% glutaraldehyde in 1× PBS. The samples were then washed in 2× saline sodium citrate and either imaged directly or stored for up to a week at 4 °C prior to imaging. For imaging, samples were mounted into a Bioptechs flow chamber, and secondary probe hybridization, step-by-step imaging of individual barcodes, and image processing were performed as described (28). Image analysis was performed as described (28).

## Supplementary Material

Supplementary File

## Data Availability

All sequencing data generated in this study have been deposited in the Gene Expression Omnibus under accession no. GSE150907. All other study data are included in the article and/or *SI Appendix*.
