# Supplementary material for: Polycomb-mediated genome architecture enables long-range spreading of H3K27 methylation
Source: Proc Natl Acad Sci U S A. 2022 May 26;119(22):e2201883119. doi: 10.1073/pnas.2201883119 (PMC9295753; doi:10.1073/pnas.2201883119)
Supplement: Supplementary File [file pnas.2201883119.sapp.pdf]

Supplementary Information for:

Polycomb-mediated Genome Architecture Enables Long-range Spreading of H3K27 Methylation

Katerina Kraft[a,1], Kathryn E. Yost[a,1], Sedona E. Murphy[b,2], Andreas Magg[c,d,2], Yicheng Long[e,f,g], M. Ryan Corces[a], Jeffrey M. Granja[a,b], Lars Wittler[h], Stefan Mundlos[c,d], Thomas R. Cech[e,f,g], Alistair N. Boettiger[i], Howard Y. Chang[a,j,3]

[a] Center for Personal Dynamic Regulomes, Stanford University School of Medicine, Stanford, CA 94305;

[b] Department of Genetics, Stanford University, Stanford, CA 94305;

[c] Research Group of Development and Disease, Max Planck Institute for Molecular Genetics, Berlin, 14195 Germany;

[d] Institute for Medical and Human Genetics, Charité Universitätsmedizin, Berlin, 10117 Germany;

[e] HHMI, University of Colorado, Boulder, CO 80309;

[f] Department of Biochemistry, University of Colorado, Boulder, CO 80309;

[g] BioFrontiers Institute, University of Colorado, Boulder, CO 80309;

[h] Department of Developmental Genetics, Max Planck Institute for Molecular Genetics, Berlin, 14195 Germany;

[i] Department of Developmental Biology, Stanford University, Stanford, CA 94305; and

[j] HHMI, Stanford University School of Medicine, Stanford, CA 94305

[1] K.K. and K.E.Y. contributed equally to this work.

[2] S. Murphy and A.M. contributed equally to this work.

[3] To whom correspondence may be addressed. Email: howchang@stanford.edu.

This PDF file includes:

Materials and Methods

Figures S1 to S8

Table S1

SI References

Materials and Methods

HiChIP data processing

HiChIP data were processed as described previously (1). Briefly, paired end reads were aligned to hg38 or mm10 genomes using the HiC-Pro pipeline (2) (version 2.11.0). Default settings were used to remove duplicate reads, assign reads to MboI restriction fragments, filter for valid interactions, and generate binned interaction matrices. The Juicer pipeline's HiCCUPS tool and FitHiChIP were used to identify loops (3, 4). Filtered read pairs from the HiC-Pro pipeline were converted into .hic format files and input into HiCCUPS using default settings. Dangling end, self-circularized, and re-ligation read pairs were merged with valid read pairs to create a 1D signal bed file. FitHiChIP was used to identify "peak-to-all" interactions at 10 kb resolution using peaks called from the one-dimensional HiChIP data. A lower distance threshold of 20 kb was used. Bias correction was performed using coverage specific bias. 1D signal bed files were converted to bigwig format for visualization using deepTools bamCoverage (version 3.3.1) with the following parameters: --bs 5 --smoothLength 105 --normalizeUsing CPM --scaleFactor 10 (5). Enrichment of 1D signal at ChIP-seq peaks was computed using deepTools computeMatrix (version 3.3.1) (5). TAD and A/B compartment annotations were obtained from a previously published mESC Hi-C dataset (6). Gene ontology enrichment at loop anchors was performed using GREAT (7) (version 4.0.4).

Virtual 4C

Virtual 4C plots were generated from dumped matrices generated with Juicebox. The Juicebox tools dump command was used to extract the chromosome of interest from the HiChIP .hic file or Hi-C .hic file from Bonev *et al.* (6) obtained from the 4D Nucleome Data Portal. E11.5 limb bud Hi-C data (8) was obtained the Gene Expression Omnibus (GEO) (GSE116794) and aligned to the mm10 genome using the HiC-Pro pipeline (2) (version 2.11.0). The interaction profile at the indicated resolution for the bin containing the anchor was then plotted in R following scaling by the total number of filtered reads in each experiment.

Cut&Tag data processing

Cut&Tag data were processed as described previously (9) with the following modifications. Paired-end reads were aligned to the mm10 genome using Bowtie2 (10) (version 2.3.4.1) with the --very-sensitive option following adapter trimming with Trimmomatic (11) (version 0.39). Reads with MAPQ values less than 10 were filtered using samtools and PCR duplicates removed using Picard's MarkDuplicates. MACS2 (12) (version 2.1.1.20160309) was used for

peak calling with the following parameters: macs2 callpeak -t input_bedpe -p 1e-5 -f BEDPE --keep-dup all -g mm -n output_file. A reproducible peak set across biological replicates was defined using the IDR framework (version 2.0.4.2). Reproducible peaks from all samples were then merged to create a union peak set. Cut&Tag signal was converted to bigwig format for visualization using deepTools bamCoverage (version 3.3.1) with the following parameters: --bs 5 --smoothLength 105 --normalizeUsing CPM --scaleFactor 10 (5). Statistical analysis was performed using DESeq2 (13) (version 1.22.2).

ChIP-seq data processing

ChIP-seq data were obtained from the Gene Expression Omnibus (GEO) using the following accession numbers: mESC EZH2 ChIP-seq (14) (GSE23943), EED cage mutant mESC H3K27me3 ChIP-seq (15) (GSE94429), mESC RAD21 and SMC1A ChIP-seq (16) (GSE137285), mESC CTCF and Ring1B ChIP-seq (6) (GSE96107), WT and *EZH2*$^{RNA-}$ iPSC EZH2 and H3K27me3 ChIP-seq (17) (GSE128135). Sequencing reads were aligned to the hg38 or mm10 genome using Bowtie2 (10) (version 2.3.4.1) with the --very-sensitive option following adapter trimming with Trimmomatic (11) (version 0.39). Reads with MAPQ values less than 10 were filtered using samtools and PCR duplicates removed using Picard's MarkDuplicates. MACS2 (12) (version 2.1.1.20160309) was used for peak calling with the following parameters: macs2 callpeak -t input_bed -f BED -n output_file --nomodel --shift 0 -q 0.01. A reproducible peak set across technical replicates was defined using the IDR framework (version 2.0.4.2). Reproducible peaks from all samples were then merged to create a union peak set. Additional processed ChIP-seq alignments and peak calls were downloaded from ENCODE from the following accessions: mESC H3K27me3 ChIP-seq (ENCSR059MBO), mESC H3K27ac ChIP-seq (ENCSR000CGQ) (18). ChIP-seq signal was converted to bigwig format for visualization using deepTools bamCoverage (version 3.3.1) with the following parameters: --bs 5 --smoothLength 105 --normalizeUsing CPM --scaleFactor 10 (5). Enrichment of ChIP-seq signal at HiChIP loop anchors was performed using Homer's annotatePeaks (19) (version 4.10). Statistical analysis was performed using DESeq2 (13) (version 1.22.2).

RNA-seq data processing

Paired-end reads were aligned to the mm10 genome using STAR (20) (version STAR_2.6.1d) following adapter trimming with Trimmomatic (11) (version 0.39). Aligned reads were filtered for uniquely mapped reads using samtools (version 1.9). Transcript counts were generate using featureCounts (21) (v1.6.4) against the Ensembl GRCm38 transcriptome (release 99). Statistical analysis was performed using DESeq2 (13) (version 1.22.2).

4C-seq data processing

4C-seq data was processed using pipe4C (22) and aligned to the hg38 genome using reading primer sequences in **SI Appendix, Table S1**.

ORCA data analysis

For all analysis in Figure 2 and supp. Figure 2, ORCA data was processed to calculate absolute distances between all barcodes for all cells. Contact frequency was calculated by calculating the fraction of cells where the probes were within a 200 nm distance. A 'cell' refers to all detected spots, with 2 spots per cell, corresponding to each allele. Absolute distances between barcodes were plotted as violin plots to show distribution of distances and wilcoxon rank-sum tests were performed to indicate significant difference between groups. Representative images were created from max projections of cells where contact between regions of interest (within 200nm) was noted.

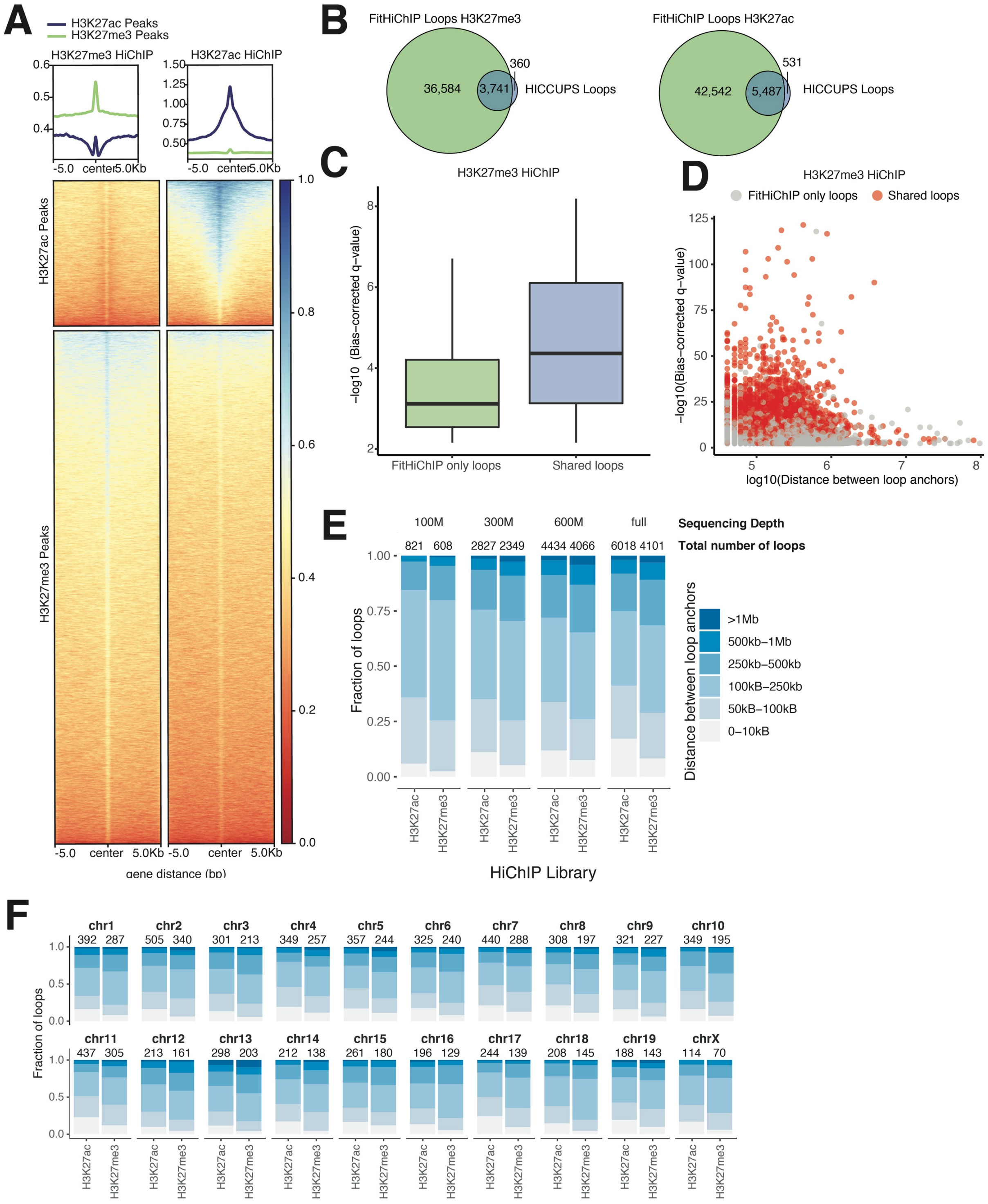

Supplementary Figure S1: Quality control for mESC H3K27me3 and H3K27ac HiChIP. (A) Enrichment of 1D HiChIP signal at H3K27me3 and H3K27ac peaks (ENCODE) (18). **(B)** Overlap between loops called by FitHiChIP and HICCUPS for mESC H3K27ac and H3K27me3 HiChIP. Loops were considered to be shared if both anchors overlap. **(C)** Boxplot of FitHiChIP bias-corrected q-values for H3K27me3 HiChIP loops called only by FitHiChIP and those shared with loops called by HICCUPS. **(D)** Scatter plot of loop distance versus bias-corrected q-value for H3K27me3 HiChIP loops called only by FitHiChIP and those shared with loops called by HICCUPS**. (E)** Bar plot of called HiChIP loops separated by distance between loop anchors for H3K27ac and H3K27me3 HiChIP in mESCs. Loops were called after downsampling to 100, 300 and 600 million reads with similar results despite fewer total loops called. **(F)** Bar plot of called HiChIP loops separated by distance between loop anchors and chromosome for H3K27ac and H3K27me3 HiChIP in mESCs, demonstrating presence of long-range H3K27me3 on all chromosomes.

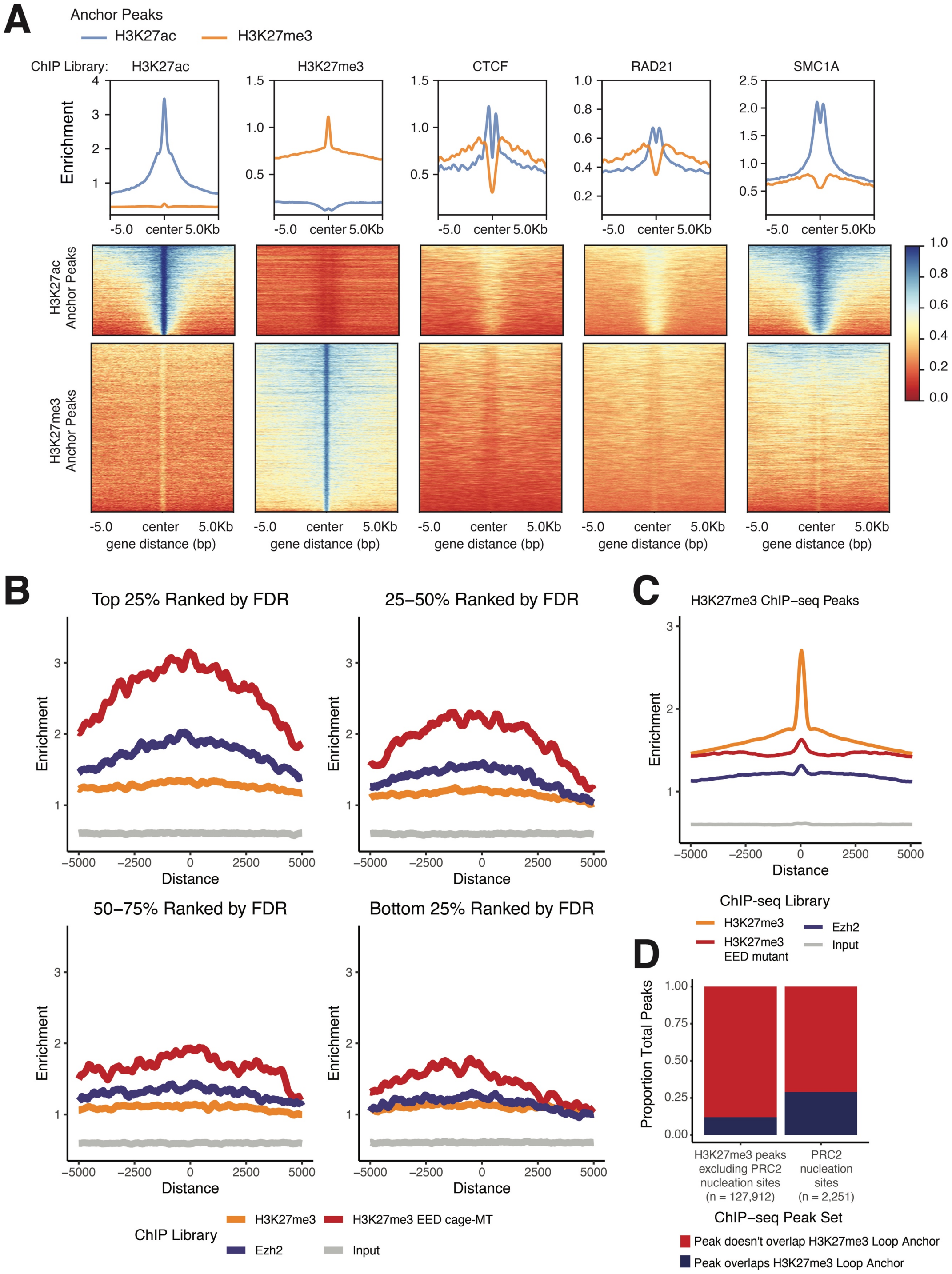

Supplementary Figure S2: Enrichment analysis at HiChIP loop anchors and comparison to ChIP-seq peaks. (A) Enrichment of mESC H3K27ac (ENCODE (18)), H3K27me3 (ENCODE (18)), CTCF (6), RAD21 (16) and SMC1A (16) ChIP-seq within a 10 kb window centered on H3K27ac (blue) or H3K27me3 (orange) ChIP-seq peaks in respective HiChIP loop anchors. **(B)** Signal enrichment for EZH2, H3K27me3 WT and EED cage-MT ChIP-seq within a 10 kb window centered on H3K27me3 HiChIP loop anchor separated by loop significance (maximum FDR reported by HICCUPS). Units of enrichment calculated as normalized ChIP-seq library depth per basepair per loop anchor. **(C)** Signal enrichment for EZH2, H3K27me3 WT and cage-EED MT ChIP-seq within a 10 kb window centered on H3K27me3 ChIP-seq peaks. Representative input control from H3K27me3 ChIP-seq included. Units of enrichment calculated as normalized ChIP-seq library depth per basepair per loop anchor. **(D)** Bar plot of PRC2 nucleation sites (EED cage-MT) and H3K27me3 peaks excluding nucleation sites colored based on overlap with H3K27me3 HiChIP loop anchors.

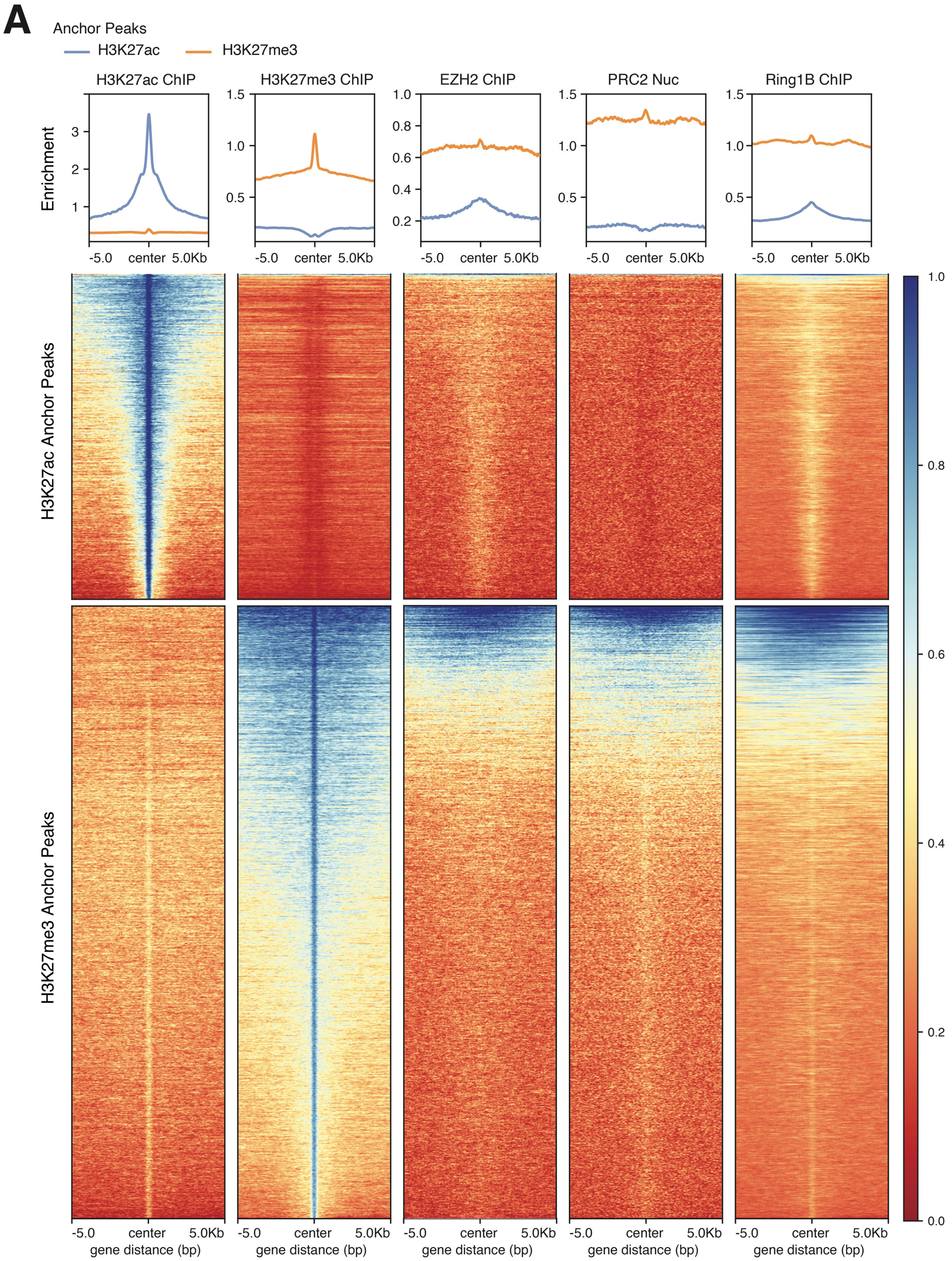

Supplementary Figure S3: PRC1 and PRC2 complex enrichment at HiChIP loop anchors. Enrichment of mESC H3K27ac (ENCODE (18)), H3K27me3 (ENCODE (18)), EZH2 (14), PRC2 nucleation/H3K27me3 EED cage MT (15), and Ring1B (6) ChIP-seq within a 10 kb window centered on H3K27ac (blue) or H3K27me3 (orange) ChIP-seq peaks in respective HiChIP loop anchors.

A

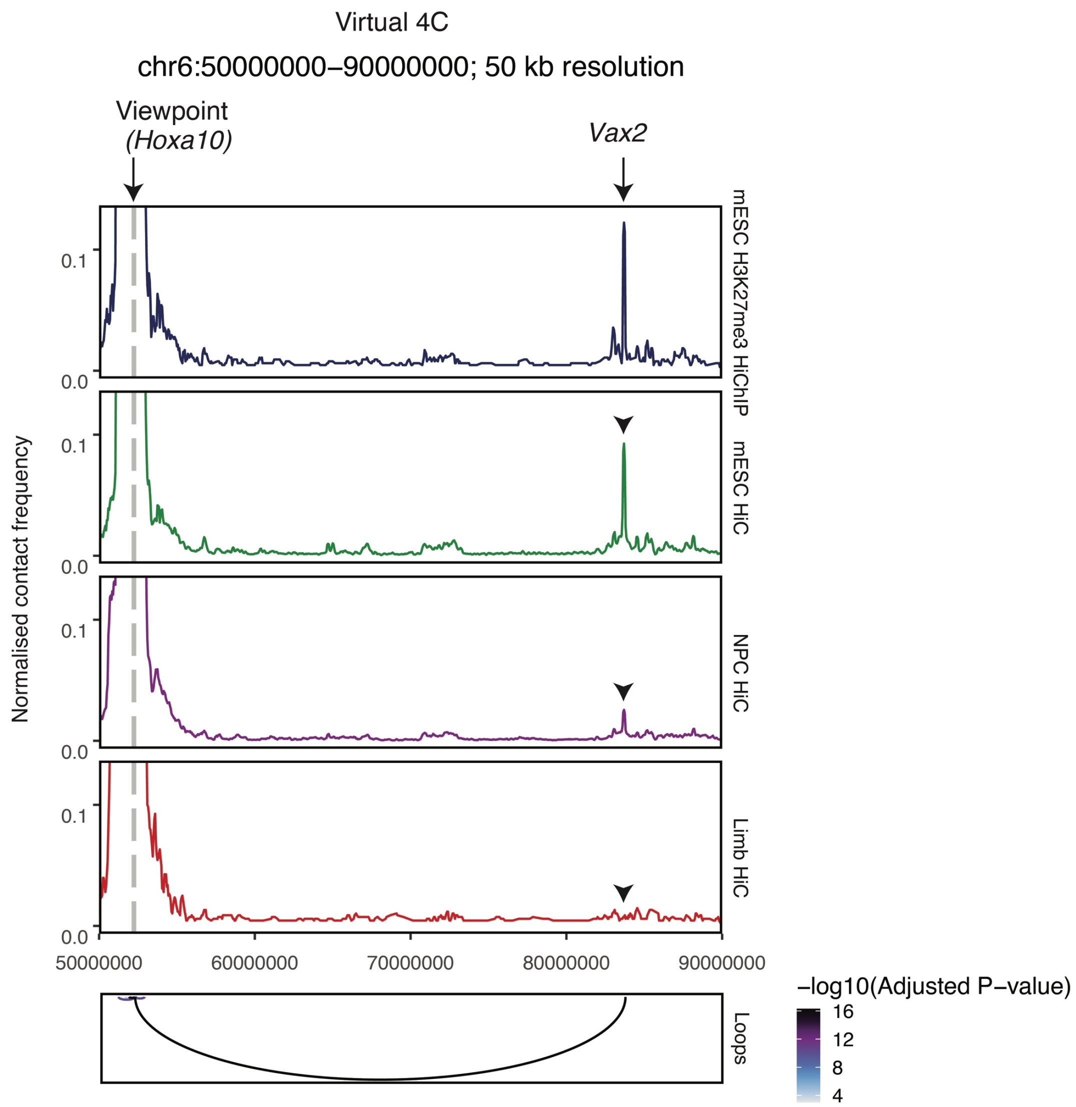

B

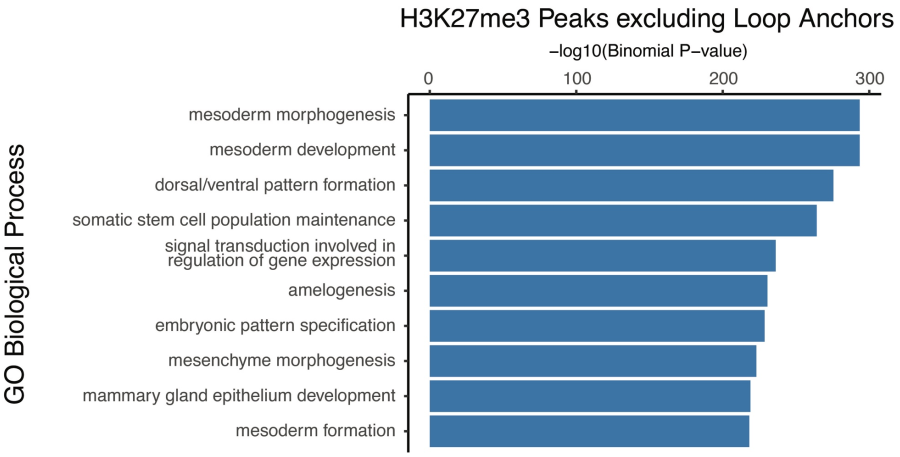

Supplementary Figure S4: Comparison of HiChIP sensitivity, loop conservation during differentiation, and ontology enrichment. (A) Virtual 4C interaction profile at the *Hoxa10* promoter for mESC H3K27me3 HiChIP (215,356,878 valid pairs; 644,288,043 total reads) (this study), mESC Hi-C (4,255,891,410 valid pairs; 7,260,480,082 total reads) (6), NPC Hi-C (5,370,123,882 valid pairs; 8,677,570,910 total reads) (6), and E11.5 limb bud Hi-C (226,690,174 valid pairs; 508,782,114 total reads) (8), scaled by number of filtered read pairs. **(B)** Gene ontology terms enriched at mESC H3K27me3 peaks excluding H3K27me3-associated loop anchors.

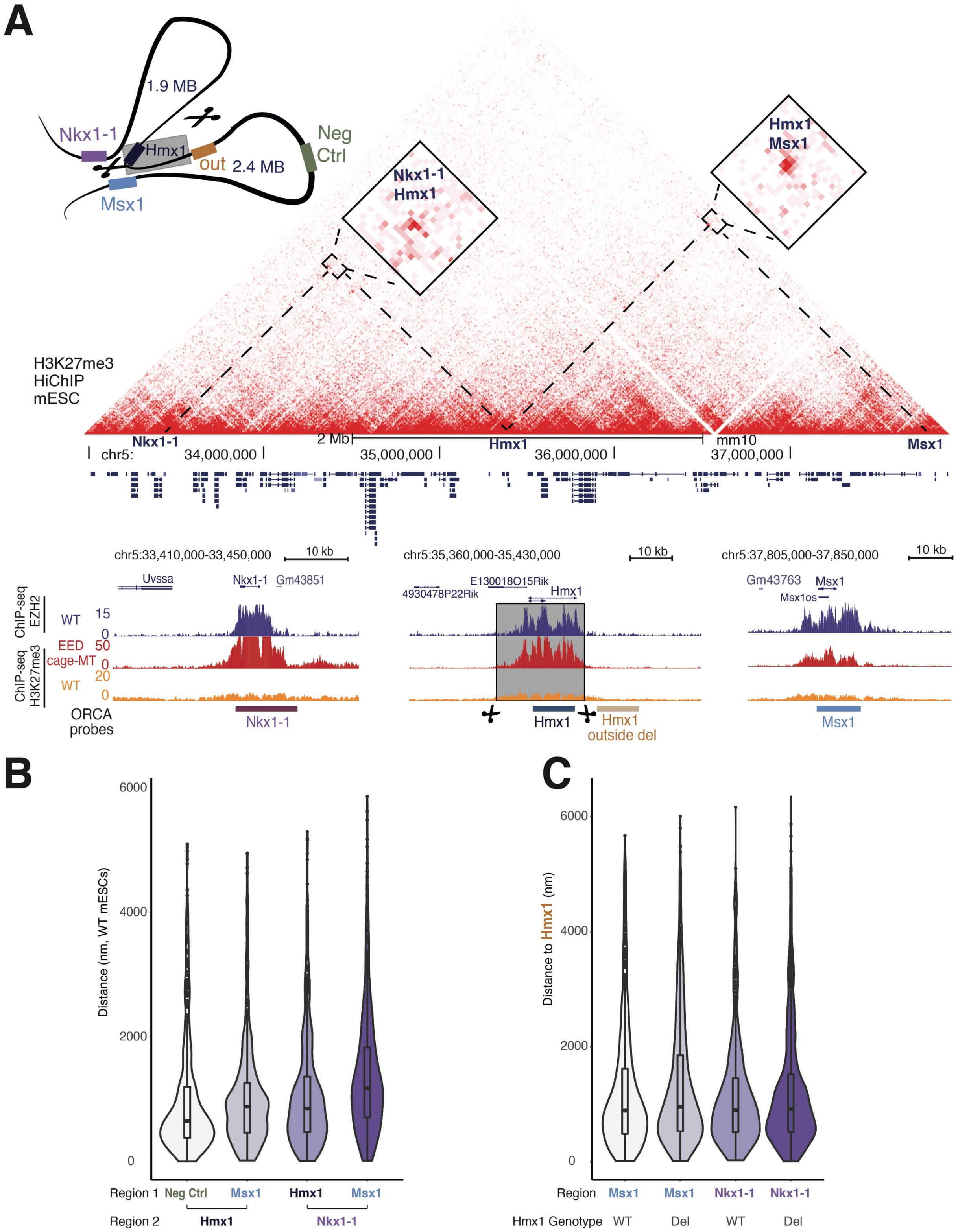

Supplementary Figure S5: Hmx1 loop anchor deletion. (A) H3K27me3 HiChIP contact matrix (10 kb resolution) at the *Nkx1-1*/*Hmx1*/*Msx1* locus in WT mESCs. ChIP-seq for WT Ezh2, H3K27me3 (WT and EED cage MT) and position of ORCA (Optical Reconstruction of Chromatin Architecture) probes is shown below. **(B and C)** Violin plots of **(B)** the distance (as measured by ORCA) of indicated regions in WT mESCs and **(C)** the distance of Hmx1 to either Msx1 or Nkx1-1 in WT and Hmx1 deletion mESCs.

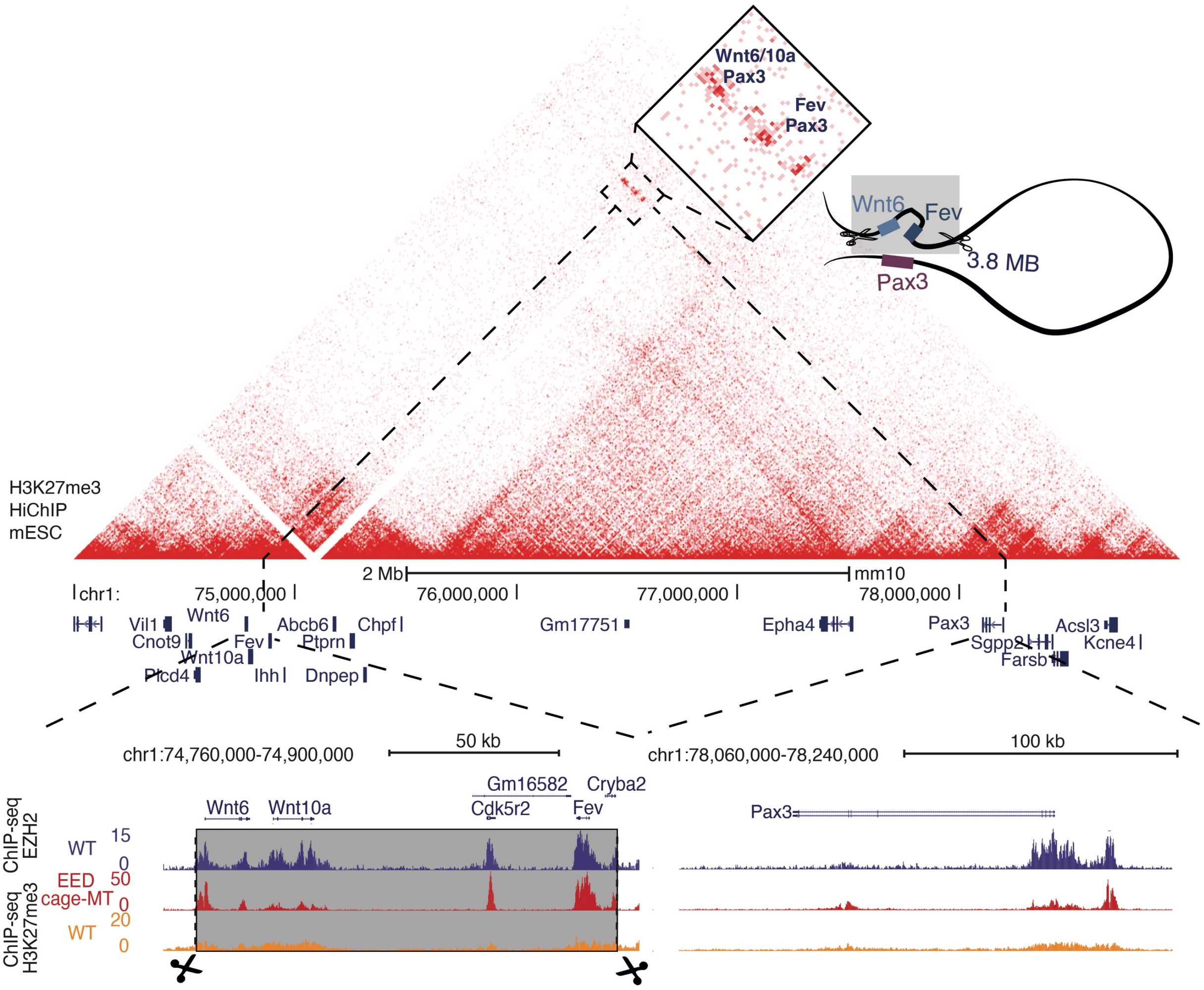

Supplementary Figure S6: Wnt6 loop anchor deletion. H3K27me3 HiChIP contact matrix (10 kb resolution) at the Wnt/Pax3 locus in WT mESCs. ChIP-seq for WT EZH2, H3K27me3 (WT and EED cage MT)

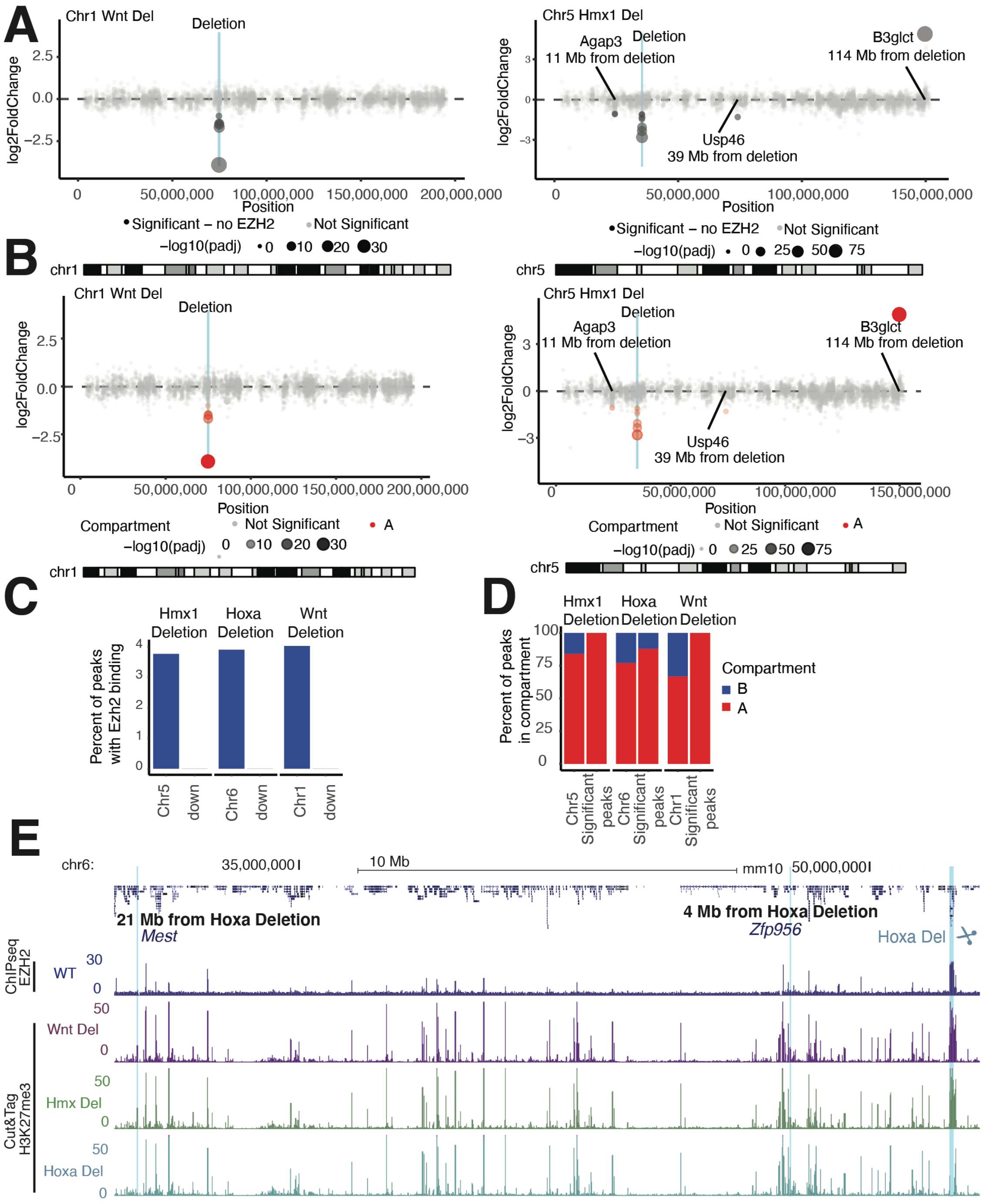

Supplementary Figure S7: Changes of H3K27me3 signal in anchor point deletions correlated with A/B compartments and EZH2 binding. (A and B) Scatter plots for the Wnt and Hmx1 anchor point deletions, demonstrating altered H3K27me Cut&Tag signal related to the genomic position on the chromosome and colored by **(A)** binding of EZH2 and **(B)** the type of compartment in which the change occurs. Log_2 fold changes and p-values (cutoff of absolute value log2FC > 1 and Benjamini-Hochberg adjusted p-value < 0.05 for significance) calculated in DESeq2 (13). **(C and D)** Bar plot for the three anchor point deletions describing the percentage of **(C)** significantly downregulated (log2FC < -2, Benjamini-Hochberg adjusted p-value < 0.05) H3K27me3 peaks with EZH2 binding in WT mESCs or **(D)** significantly altered H3K27me3 peaks (absolute value log2FC > 1, Benjamini-Hochberg adjusted p-value < 0.05) colored by A/B compartment in which the H2K27me3 peak is located. Distributions of EZH2 binding and A/B compartments for all H3K27me3 peaks on that chromosome included for comparison. **(E)** Changes on chromosome 6 depicted by H3K27me3 Cut&Tag signal track, blue marked regions indicate significantly altered regions in mutant Hoxa Del. Zoom in of these regions are shown in Figure 3C.

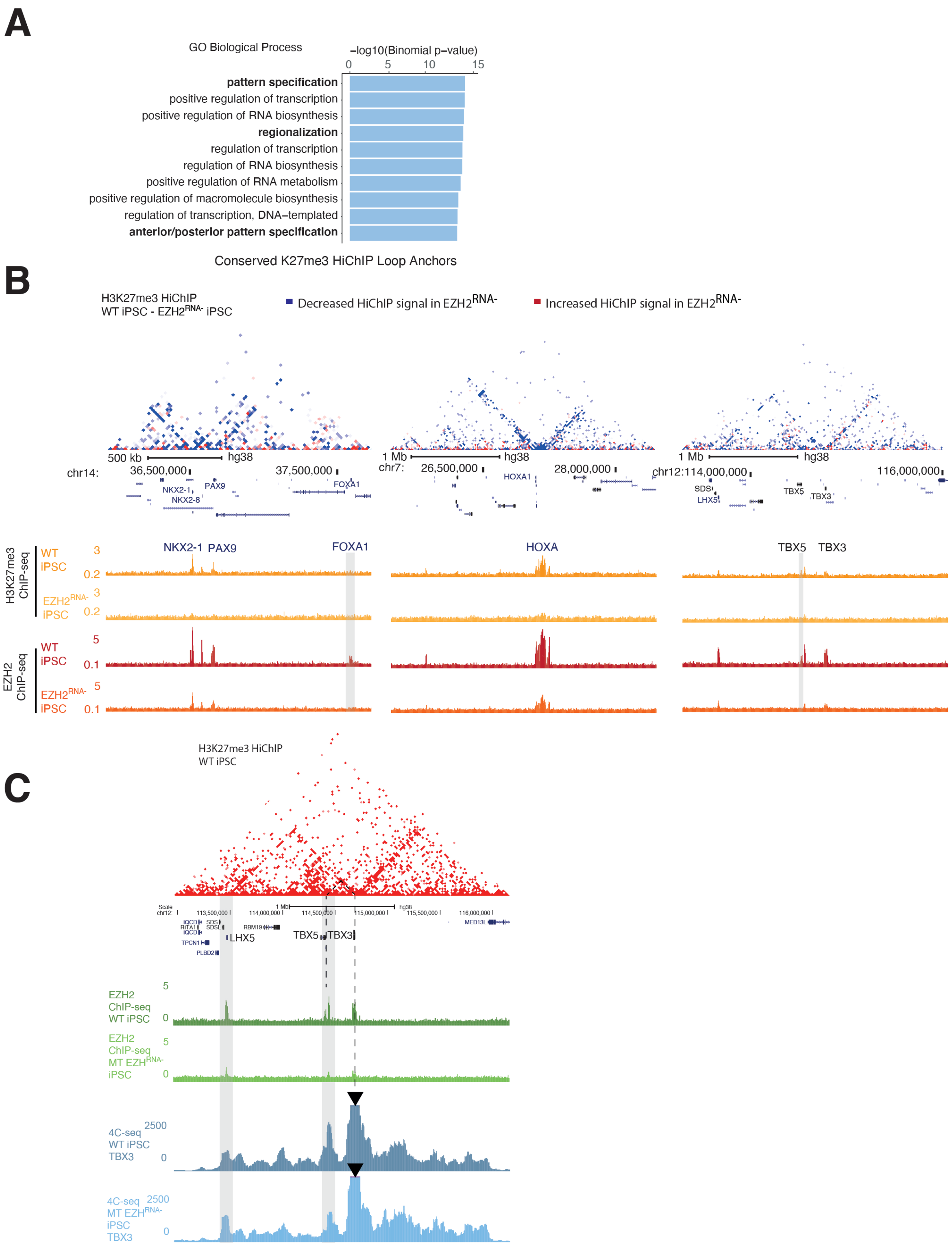

Supplementary Figure S8: Altered polycomb binding due to loss of RNA binding by EZH2 influences the chromatin conformation and H3K27me3 spreading at different developmental loci. (A) Gene ontology terms enriched at conserved H3K27me3-associated loop anchors between mESCs and iPSCs**. (B)** iPSC H3K27me3 HiChIP subtraction contact matrices (WT – *EZH2^{RNA-}*, 20 kb resolution) for three representative developmental loci, with corresponding ChIP-seq tracks of H3K27me3 and EZH2 below. Blue color in the subtraction maps corresponds to decreased signal in *EZH2^{RNA-}* iPSCs. **(C)** H3K27me3 HiChIP contact matrix (20 kb resolution) showing the 3D chromatin configuration at the *TBX3*/*TBX5* locus in WT human iPSCs. EZH2 ChIP-seq and 4C-seq at *TBX3* viewpoint in WT and *EZH2^{RNA-}* iPSCs is shown below (n=1). Reduced contact with *TBX5* accompanied by reduced EZH2 binding highlighted.

**ORCA probes**			
Primary probes	Coordinates mm10	Fiducial probes	Coordinates mm10
Hmx1_inside_del	chr5:35,390,000-35,400,000	Fid_chr5	chr5:35,422,516-35,612,516
Msx1os	chr5:37,820,000-37,830,000		
Hmx1_outside_del	chr5:35,402,516-35,412,516		
Nkx1-1	chr5:33,430,000-33,440,000		
Pp2r2c (negative cntr)	chr5:36,910,000-36,920,000		
Hoxa9_inside_del	chr6:52225000-52235000	Fid_chr6	chr6:51,808,339-51,908,339
Vax2os	chr6:83710000-83700000		
Hoxa_outside_del	chr6:52120000-52130000		
(negative cntr)	chr6:68900000-68910000		
**CRISPR-Cas9 deletions in mESC**			
Mutant	sgRNA target sequence		CRISPR breakpoint (mm10)
Wnt-Del	Wnt-cen	ACATAAATGGGGAGCACCTA	Chr1: 74,769,315-74,901,102
	Wnt-tel	GAGCAAACGGGCGCCCGGAC	
Hmx1-Del	Hmx1-cen	CGTTGATCGACTGTCCTCAC	Chr5: 35,381,176-35,402,334
	Hmx1-tel	GTTGATGCTGCACACCGACA	
HoxA-Del	HoxA-cen	ACAAAACCACGGTTACTAGC	Chr6: 52,161,884-52,272,698
	HoxA-tel	CCACTTCACAGGTCGTTATG	
Genotyping PCR primers		CNV qPCR primers	
Wnt-F1	GGGGGAGTTAAGGGTGTGAT	q_Wnt-1-F	ACCACTTGGAGGAGAACACTTGC
Wnt-R1	CCCCCACCTTTCCTCTAAAC	q_Wnt-1-R	GCAAACTACCCAGCCCTACCTCT
Wnt-F2	GGTGTCTGGTGGATGAGCTT	q_Wnt-2-F	TGCCGGTCCCTTTCTAAGACATA
Wnt-R2	GATGTTCTGCCTTCGCTAGG	q_Wnt-2-R	CCCTTTTCTTTGCTGGCCTAGAT
Hmx1-F1	CCAAAAGAGGGAAGATGCAA	q_Hmx1-1-F	TAGTGCTTGGCAGCCTTACTTCC
Hmx1-R1	CGCCTCAATTTTCTCTCAGC	q_Hmx1-1-R	CTACACGGAGGAGAGCAGAGAGG

Hmx1-F2	GATGTGGATGAACGTGCTTG	q_Hmx1-2-F	AGAGTGGGCTCTGCATAGGTTTG
Hmx1-R2	ATCCACCCACACTCAAGGAG	q_Hmx1-2-R	CTCAGCTGGACATTTTGGAACCT
HoxA-F1	TGGGAGGAGACAAAAGAAGG	q_HoxA-1-F	TCCCAAATACCAGTTCTGGAGGA
HoxA-R1	AGCCCAAGCTTTTGAGTGTG	q_HoxA-1-R	CTTGGGTTTCCAAGCCTCTAGGT
HoxA-F2	CCCAAGGTCAAGAAAGTCCA	q_HoxA-2-F	TAGAGGTTCACAAGCCCAGAAGG
HoxA-R2	TCTGAGCACCAAACCCTCTC	q_HoxA-2-R	GGTGAAGGTGCAGCATCTCTTTT
**4C-seq**			
4C-seq primer	Restriction enzymes HindIII 1st/ NlaIII 2nd		
Viewpoint	reading primer postion (hg38)	Reading primer with adaptor	Non-reading primer with adaptor
NKX2-1	chr14:36,522,817	TCGTCGGCAGCGTCAGATGTGTATAAGAGACAGATCTGTATCTCCAAGCTT	GTCTCGTGGGCTCGGAGATGTGTATAAGAGACAGTCTTCCTTTTACTCAGGTC
PAX9	chr14:36,658,809	TCGTCGGCAGCGTCAGATGTGTATAAGAGACAGTGCTGGGGTCTAAAGCTT	GTCTCGTGGGCTCGGAGATGTGTATAAGAGACAGGTTTTCAACAGCGGAATTGC
TBX3	chr12:114,685,710	TCGTCGGCAGCGTCAGATGTGTATAAGAGACAGTTCCTGAGATGAAAGCTT	GTCTCGTGGGCTCGGAGATGTGTATAAGAGACAGTTGTCCAGATCCTCCATT

Supplementary Table S1: ORCA probes, sgRNAs, and primers used in this study.

References:

1. Mumbach MR, Rubin AJ, Flynn RA, Dai C, Khavari PA, Greenleaf WJ, et al. HiChIP: efficient and sensitive analysis of protein-directed genome architecture. Nat Methods. 2016;13(11):919-22.
2. Servant N, Varoquaux N, Lajoie BR, Viara E, Chen CJ, Vert JP, et al. HiC-Pro: an optimized and flexible pipeline for Hi-C data processing. Genome Biol. 2015;16:259.
3. Rao SS, Huntley MH, Durand NC, Stamenova EK, Bochkov ID, Robinson JT, et al. A 3D Map of the Human Genome at Kilobase Resolution Reveals Principles of Chromatin Looping. Cell. 2014.
4. Bhattacharyya S, Chandra V, Vijayanand P, Ay F. Identification of significant chromatin contacts from HiChIP data by FitHiChIP. Nat Commun. 2019;10(1):4221.
5. Ramirez F, Ryan DP, Gruning B, Bhardwaj V, Kilpert F, Richter AS, et al. deepTools2: a next generation web server for deep-sequencing data analysis. Nucleic Acids Res. 2016;44(W1):W160-5.
6. Bonev B, Mendelson Cohen N, Szabo Q, Fritsch L, Papadopoulos GL, Lubling Y, et al. Multiscale 3D Genome Rewiring during Mouse Neural Development. Cell. 2017;171(3):557-72 e24.
7. McLean CY, Bristor D, Hiller M, Clarke SL, Schaar BT, Lowe CB, et al. GREAT improves functional interpretation of cis-regulatory regions. Nat Biotechnol. 2010;28(5):495-501.
8. Kraft K, Magg A, Heinrich V, Riemenschneider C, Schopflin R, Markowski J, et al. Serial genomic inversions induce tissue-specific architectural stripes, gene misexpression and congenital malformations. Nat Cell Biol. 2019;21(3):305-10.
9. Kaya-Okur HS, Wu SJ, Codomo CA, Pledger ES, Bryson TD, Henikoff JG, et al. CUT&Tag for efficient epigenomic profiling of small samples and single cells. Nat Commun. 2019;10(1):1930.
10. Langmead B, Salzberg SL. Fast gapped-read alignment with Bowtie 2. Nat Methods. 2012;9(4):357-9.
11. Bolger AM, Lohse M, Usadel B. Trimmomatic: a flexible trimmer for Illumina sequence data. Bioinformatics. 2014;30(15):2114-20.
12. Zhang Y, Liu T, Meyer CA, Eeckhoute J, Johnson DS, Bernstein BE, et al. Model-based analysis of ChIP-Seq (MACS). Genome Biol. 2008;9(9):R137.
13. Love MI, Huber W, Anders S. Moderated estimation of fold change and dispersion for RNA-seq data with DESeq2. Genome Biol. 2014;15(12):550.
14. Marks H, Kalkan T, Menafra R, Denissov S, Jones K, Hofemeister H, et al. The transcriptional and epigenomic foundations of ground state pluripotency. Cell. 2012;149(3):590-604.
15. Oksuz O, Narendra V, Lee CH, Descostes N, LeRoy G, Raviram R, et al. Capturing the Onset of PRC2-Mediated Repressive Domain Formation. Mol Cell. 2018;70(6):1149-62 e5.
16. Justice M, Carico ZM, Stefan HC, Dowen JM. A WIZ/Cohesin/CTCF Complex Anchors DNA Loops to Define Gene Expression and Cell Identity. Cell Rep. 2020;31(2):107503.
17. Long Y, Hwang T, Gooding AR, Goodrich KJ, Rinn JL, Cech TR. RNA is essential for PRC2 chromatin occupancy and function in human pluripotent stem cells. Nat Genet. 2020.
18. Yue F, Cheng Y, Breschi A, Vierstra J, Wu W, Ryba T, et al. A comparative encyclopedia of DNA elements in the mouse genome. Nature. 2014;515(7527):355-64.
19. Heinz S, Benner C, Spann N, Bertolino E, Lin YC, Laslo P, et al. Simple combinations of lineage-determining transcription factors prime cis-regulatory elements required for macrophage and B cell identities. Mol Cell. 2010;38(4):576-89.

20. Dobin A, Davis CA, Schlesinger F, Drenkow J, Zaleski C, Jha S, et al. STAR: ultrafast universal RNA-seq aligner. Bioinformatics. 2013;29(1):15-21.
21. Liao Y, Smyth GK, Shi W. featureCounts: an efficient general purpose program for assigning sequence reads to genomic features. Bioinformatics. 2014;30(7):923-30.
22. Krijger PHL, Geeven G, Bianchi V, Hilvering CRE, de Laat W. 4C-seq from beginning to end: A detailed protocol for sample preparation and data analysis. Methods. 2020;170:17-32.
